# The Time Scale of Evolutionary Innovation

**DOI:** 10.1371/journal.pcbi.1003818

**Published:** 2014-09-11

**Authors:** Krishnendu Chatterjee, Andreas Pavlogiannis, Ben Adlam, Martin A. Nowak

**Affiliations:** 1 IST Austria, Klosterneuburg, Austria; 2 Program for Evolutionary Dynamics, Department of Organismic and Evolutionary Biology, Department of Mathematics, Harvard University, Cambridge, Massachusetts, United States of America; ETH Zurich, Switzerland

## Abstract

A fundamental question in biology is the following: what is the time scale that is needed for evolutionary innovations? There are many results that characterize single steps in terms of the fixation time of new mutants arising in populations of certain size and structure. But here we ask a different question, which is concerned with the much longer time scale of evolutionary trajectories: how long does it take for a population exploring a fitness landscape to find target sequences that encode new biological functions? Our key variable is the length, 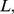 of the genetic sequence that undergoes adaptation. In computer science there is a crucial distinction between problems that require algorithms which take polynomial or exponential time. The latter are considered to be intractable. Here we develop a theoretical approach that allows us to estimate the time of evolution as function of 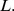 We show that adaptation on many fitness landscapes takes time that is exponential in 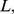 even if there are broad selection gradients and many targets uniformly distributed in sequence space. These negative results lead us to search for specific mechanisms that allow evolution to work on polynomial time scales. We study a regeneration process and show that it enables evolution to work in polynomial time.

## Author Summary

Evolutionary adaptation can be described as a biased, stochastic walk of a population of sequences in a high dimensional sequence space. The population explores a fitness landscape. The mutation-selection process biases the population towards regions of higher fitness. In this paper we estimate the time scale that is needed for evolutionary innovation. Our key parameter is the length of the genetic sequence that needs to be adapted. We show that a variety of evolutionary processes take exponential time in sequence length. We propose a specific process, which we call ‘regeneration processes’, and show that it allows evolution to work on polynomial time scales. In this view, evolution can solve a problem efficiently if it has solved a similar problem already.

## Introduction

Our planet came into existence 4.6 billion years ago. There is clear chemical evidence for life on earth 3.5 billion years ago [1], [2]. The evolutionary process generated procaria, eucaria and complex multi-cellular organisms. Throughout the history of life, evolution had to discover sequences of biological polymers that perform specific, complicated functions. The average length of bacterial genes is about 1000 nucleotides, that of human genes about 3000 nucleotides. The longest known bacterial gene contains more than 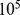 nucleotides, the longest human gene more than 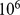. A basic question is what is the time scale required by evolution to discover the sequences that perform desired functions. While many results exist for the fixation time of individual mutants [3]–[15], here we ask how the time scale of evolution depends on the length 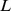 of the sequence that needs to be adapted. We consider the crucial distinction of polynomial versus exponential time [16]–[18]. A time scale that grows exponentially in 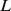 is infeasible for long sequences.

Evolutionary dynamics operates in sequence space, which can be imagined as a discrete multi-dimensional lattice that arises when all sequences of a given length are arranged such that nearest neighbors differ by one point mutation [19]. For constant selection, each point in sequence space is associated with a non-negative fitness value (reproductive rate). The resulting fitness landscape is a high dimensional mountain range. Populations explore fitness landscapes searching for elevated regions, ridges, and peaks [20]–[27].

A question that has been extensively studied is how long does it take for existing biological functions to improve under natural selection. This problem leads to the study of adaptive walks on fitness landscapes [15], [20], [21], [28], [29]. In this paper we ask a different question: how long does it take for evolution to discover a new function? More specifically, our aim is to estimate the expected discovery time of new biological functions: how long does it take for a population of reproducing organisms to discover a biological function that is not present at the beginning of the search. We will discuss two approximations for rugged fitness landscapes. We also discuss the significance of clustered peaks.

We consider an alphabet of size four, as is the case for DNA and RNA, and a nucleotide sequence of length 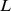. We consider a population of size 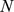, which reproduces asexually. The mutation rate, 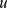, is small: individual mutations are introduced and evaluated by natural selection and random drift one at a time. The probability that the evolutionary process moves from a sequence 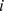 to a sequence 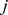, which is at Hamming distance one from 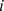, is given by 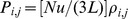, where 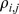 is the fixation probability of sequence 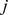 in a population consisting of sequence 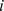. In the special case of a flat fitness landscape, we have 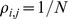, and 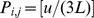. Thus we have an evolutionary random walk, where each step is a jump to a neighboring sequence of Hamming distance one.

## Results

Consider a high-dimensional sequence space. A particular biological function can be instantiated by some of the sequences. Each sequence 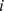 has a fitness value 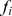, which measures the ability of the sequence 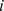 to encode the desired function. Biological fitness landscapes are typically expected to have many peaks [29]–[31]. They can be highly rugged due to epistatic effects of mutations [32]–[34]. They can also contain large regions or networks of neutrality [20], [21]. Empirical studies of short RNA sequences have revealed that the underlying fitness landscape has low peak density [35]: around 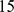 peaks in 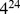 sequences.

**Figure 1:**
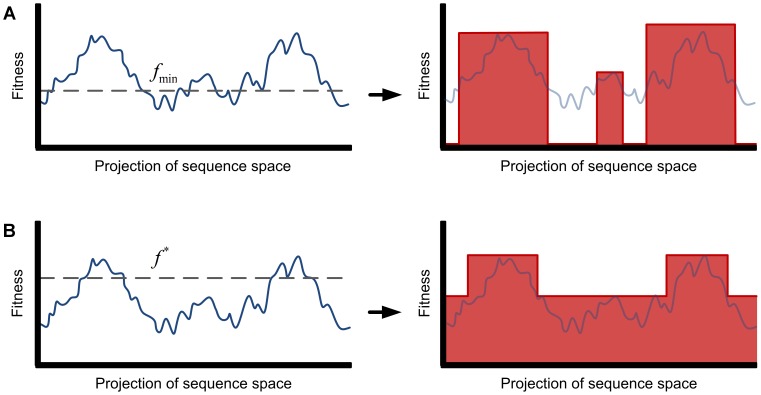
Approximations of a highly rugged fitness landscape by broad peaks and neutral regions. The figures depict examples of highly rugged fitness landscapes where the sequence space has been projected in one dimension. (A) Sequences with fitness below some level 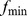 are functionally very different to the desired function, and selection cannot act upon them. All other sequences are considered as targets. The fitness landscape is approximated by a step function: if 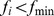, then 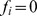, otherwise 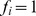. (B) Local maxima below the desired fitness threshold 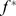 are known to slow down the evolutionary random walk towards sequences that attain fitness at least 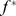. We approximate the fitness landscape by broad peaks and neutral regions by increasing the fitness of every sequence that belongs in a mountain range with fitness below 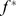 to the maximal local maxima 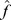 below 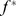. Note that the target set starts from the upslope of a mountain range whose peak exceeds 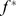.

For the purpose of estimating the expected discovery time we can approximate the fitness landscape with a binary step function over the sequence space. We discuss two different approximations (Figure 1). For the first approximation, we consider the scenario where fitness values below some threshold, 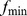, have negligible contribution; those sequences do not instantiate the desired function (either not at all or only below the minimum level that could be detected by natural selection). We approximate the rugged fitness landscape as follows: if 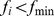 then 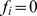; if 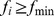 then 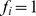. The set of sequences with 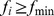 constitutes the target set, and the remaining fitness landscape is neutral.

The second approximation works as follows. Consider the evolutionary process exploring a rugged fitness landscape where the goal is to attain a fitness level 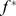. Local maxima below 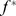 slow down the evolutionary process to attain 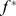, because the evolutionary walk might get stuck in those local maxima. In order to derive lower bounds for the expected discovery time, the rugged fitness landscape can be approximated as follows. Let 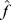 be the fitness value of the highest local maximum below 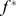. Then for every sequence in a mountain range with a local maximum below 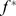 we assign the fitness value 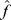. The mountain ranges with local maxima above 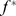 are the target sequences. Note that the target set includes sequences that start at the upslope of mountain ranges with peaks above 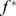. Thus, again we obtain a fitness landscape with clustered targets and neutral region, where the neutral region consists of all sequences whose fitness values have been assigned to 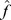. The two approximations are illustrated in Figure 1. For 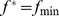 the second approximation generates larger target areas than the first approximation and is therefore more lenient.

Our key results for estimating the discovery time can now be formulated for binary fitness landscapes, but they apply to any type of rugged landscape using one of the two approximations. We note that our methods can also be applied for certain non-binary fitness landscapes, and an example of a fitness landscape with a large gradient arising from multiplicative fitness effects is discussed in Sections 6 and 7 of Text S1.

We now present our main results in the following order. We first estimate the discovery time of a single search aiming to find a single broad peak. Then we study multiple simultaneous searches for a single broad peak. Finally, we consider multiple broad peaks that are uniformly randomly distributed in sequence space.

We first study a broad peak of target sequences described as follows: consider a specific sequence; any sequence within a certain Hamming distance of that sequence belongs to the target set. Specifically, we consider that the evolutionary process has succeeded, if the population discovers a sequence that differs from the specific sequence in no more than a fraction 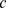 of positions. We refer to the specific sequence as the target center and 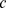 as the width (or radius) of the peak. For example, if 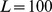 and 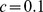, then the target center is surrounded by a cloud of approximately 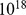 sequences. For a single broad peak with width 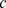, the target set contains at least 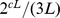 sequences, which is an exponential function of 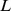. The fitness landscape outside the broad peak is flat. We refer this binary fitness landscape as a broad peak landscape. The population needs to discover any one of the target sequences in the broad peak, starting from some sequence that is not in the broad peak. We establish the following result.

### 

#### Theorem 1

*Consider a single search exploring a broad peak landscape with width* 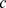 *and mutation rate* 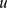 *. The following assertions hold*:

- *if* 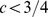 *, then there exists* 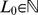 *such that for all sequence spaces of sequence length* 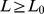 *, the expected discovery time is at least* 
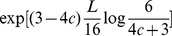
 *;*
- *if* 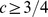 *, then for all sequence spaces of sequence length* 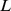 *, the expected discovery time is at most* 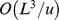 *.*

Our result can be interpreted as follows (see Theorem S2 and Corollary S2 in Text S1): (i) If 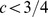, then the expected discovery time is exponential in 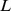; and (ii) if 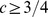, then the expected discovery time is polynomial in 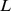. Thus, we have derived a *strong dichotomy* result which shows a sharp transition from polynomial to exponential time depending on whether a specific condition on 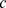 does or does not hold.

**Figure 2:**
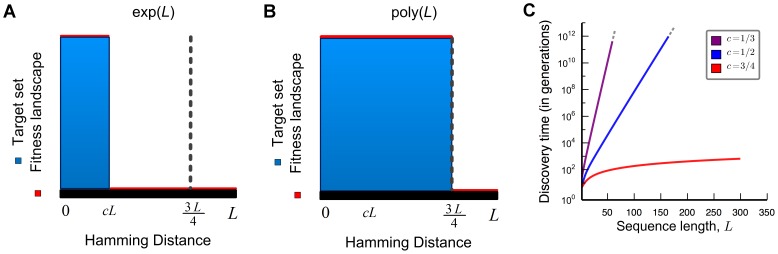
Broad peak with different fitness landscapes. For the broad peak there is a specific sequence, and all sequences that are within Hamming distance 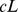 are part of the target set. The fitness landscape is flat outside the broad peak. (A) If the width of the broad peak is 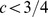, then the expected discovery time is exponential in sequence length, 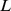. (B) If the width of the broad peak is 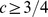, then the expected discovery time is polynomial in sequence length, 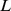. (C) Numerical calculations for broad peak fitness landscapes. We observe exponential expected discovery time for 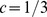 and 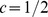, whereas polynomial expected discovery time for 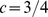.

For the four letter alphabet most random sequences have Hamming distance 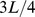 from the target center. If the population is further away than this Hamming distance, then random drift will bring it closer. If the population is closer than this Hamming distance, then random drift will push it further away. This argument constitutes the intuitive reason that 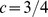 is the critical threshold. If the peak has a width of less than 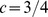, then we prove that the expected discovery time by random drift is exponential in the sequence length 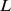 (see Figure 2). This result holds for any population size, 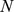, as long as 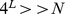, which is certainly the case for realistic values of 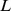 and 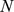. In the Text S1 we also present a more general result, where along with a single broad peak, instead of a flat landscape outside the peak we consider a multiplicative fitness landscape and establish a sharp dichotomy result that generalizes Theorem 1 (see Corollary S2 in Text S1).

#### Remark 1

*We highlight two important aspects of our results.*

1. *First, when we establish exponential lower bounds for the expected discovery time, then these lower bounds hold even if the starting sequence is only a few steps away from the target set.*
2. *Second, we present strong dichotomy results, and derive mathematically the most precise and strongest form of the boundary condition.*

Let us now give a numerical example to demonstrate that exponential time is intractable. Bacterial life on earth has been around for at least 3.5 billion years, which correspond to 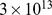 hours. Assuming fast bacterial cell division of 20–30 minutes on average we have at most 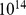 generations. The expected discovery time for a sequence of length 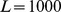 with a very large broad peak of 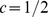 is approximately 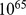 generations; see Table 1.

**Table 1:** Numerical data for discovery time in flat fitness landscapes.

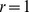	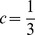	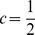	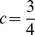
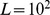	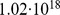	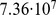	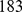
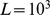	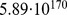	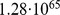	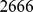

Numerical data for the discovery time of broad peaks with width 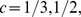 and 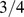 embedded in flat fitness landscapes. First the discovery time is computed for small values of 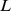 as shown in Figure 2(C). Then the exponential growth is extrapolated to 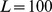 and 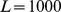, respectively. We show the discovery times for 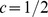, and 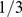. For 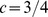 the values are polynomial in 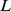.

If individual evolutionary processes cannot find targets in polynomial time, then perhaps the success of evolution is based on the fact that many populations are searching independently and in parallel for a particular adaptation. We prove that multiple, independent parallel searches are not the solution of the problem, if the starting sequence is far away from the target center. Formally we show the following result.

#### Theorem 2

*In all cases where the lower bound on the expected discovery time is exponential, for all polynomials* 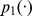 *,* 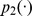 *and* 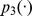 *, for any starting sequence with Hamming distance at least* 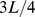 *from the target center, the probability for any one out of* 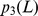 *independent multiple searches to reach the target set within* 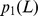 *steps is at most* 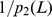 *.*

If an evolutionary process takes exponential time, then polynomially many independent searches do not find the target in polynomial time with reasonable probability (for details see Theorem S5 in the Text S1). We also show an informal and approximate calculation of the success probability for 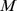 independent searches, as follows: if the expected discovery time is exponential (say, 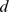), then the probability that all 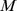 independent searches fail upto 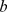 steps is at least 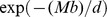 (i.e., the success probability within 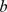 steps of any of the searches is at most 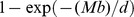), when the starting sequence is far away from the target center. In such a case, one could quickly exhaust the physical resources of an entire planet. The estimated number of bacterial cells [36] on earth is about 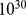. To give a specific example let us assume that there are 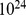 independent searches, each with population size 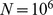. The probability that at least one of those independent searches succeeds within 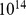 generations for sequence length 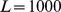 and broad peak of 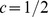 is less than 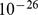.

In our basic model, individual mutants are evaluated one at a time. The situation of many mutant lineages evolving in parallel is similar to the multiple searches described above. As we show that whenever a single search takes exponential time, multiple independent searches do not lead to polynomial time solutions, our results imply intractability for this case as well.

We now explore the case of multiple broad peaks that are uniformly and randomly distributed. Consider that there are 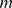 target centers. Around each target center there is a selection gradient extending up to a distance 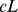. Formally we can consider any fitness function 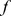 that assigns zero fitness to a sequence whose Hamming distance exceeds 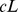 from all the target centers, which in particular is subsumed by considering the multiple broad peaks where around each center we consider a broad peak of target set with peak width 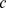. We establish the following result:

#### Theorem 3

*Consider a single search under the multiple broad peak fitness landscape of* 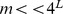 *target centers chosen uniformly at random, with peak width at most* 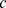 *for each center and* 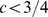 *. Then with high probability, the expected discovery time of the target set is at least* 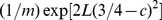 *.*

**Figure 3:**
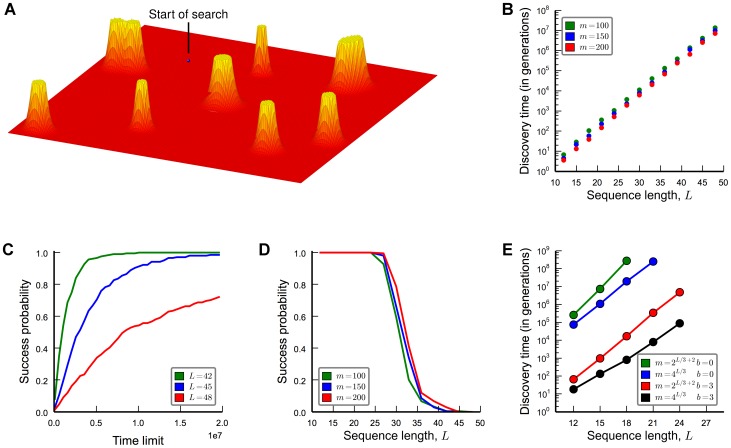
The search for randomly, uniformly distributed targets in sequence space. (A) The target set consists of 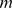 random sequences; each one of them is surrounded by a broad peak of width up to 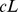. The figure shows a pictorial illustration where the 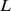-dimensional sequence space is projected onto two dimensions. From a randomly chosen starting sequence outside the target set, the expected discovery time is at least 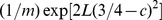, which can be exponential in 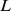. (B) Computer simulations showing the average discovery time of 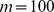, 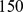, and 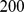 targets, with 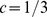. We observe exponential dependency on 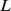. The discovery time is averaged over 200 runs. (C) Success probability estimated as the fraction of the 200 searches that succeed in finding one of the target sequences within 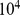 generations. The success probability drops exponentially with 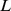. (D) Success probability as a function of time for 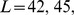 and 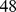. (E) Discovery time for a large number of randomly generated target sequences. Either 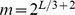 or 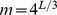 sequences were generated. For 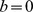 and 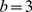 the target set consists of balls of Hamming distance 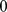 and 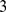 (respectively) around each sequence. The figure shows the average discovery time of 100 runs. As expected we observe that the discovery time grows exponentially with sequence length, 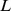.

Whether or not the function 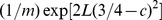 is exponential in 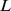 depends on how 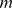 changes with 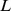. But even if we assume exponentially many broad peak centers, 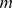, with peak width 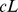 where 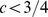, we need not obtain polynomial time (Figure 3 and Theorem S6 in Text S1).

It is known that recombination may accelerate evolution on certain fitness landscapes [28], [37]–[39], and recombination may also slow down evolution on other fitness landscapes [40]. Recombination, however, reduces the discovery time only by at most a linear factor in sequence length [28], [37], [38], [41], [42]. A linear or even polynomial factor improvement over an exponential function does not convert the exponential function into a polynomial one. Hence, recombination can make a significant difference only if the underlying evolutionary process without recombination already operates in polynomial time.

**Figure 4:**
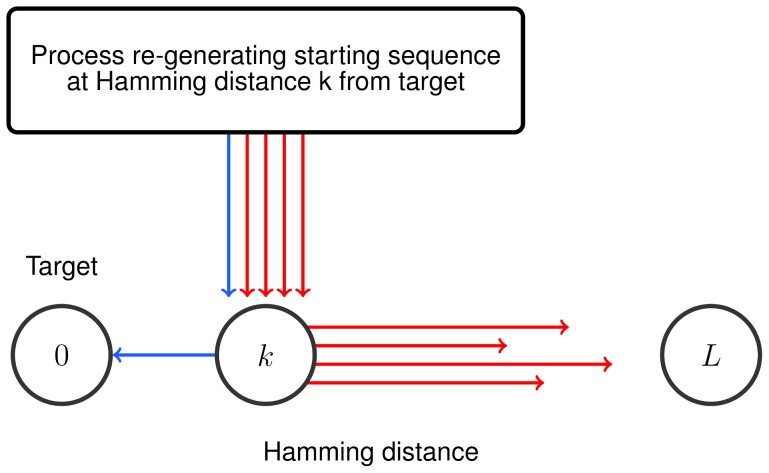
Regeneration process. Gene duplication (or possibly some other process) generates a steady stream of starting sequences that are a constant number 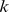 of mutations away from the target. Many searches drift away from the target, but some will succeed in polynomially many steps. We prove that 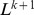 searches ensure that with high probability some search succeed in polynomially many steps.

What are then adaptive problems that can be solved by evolution in polynomial time? We propose a “regeneration process”. The basic idea is that evolution can solve a new problem efficiently, if it is has solved a similar problem already. Suppose gene duplication or genome rearrangement can give rise to starting sequences that are at most 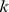 point mutations away from the target set, where 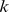 is a number that is independent of 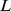. It is important that starting sequences can be regenerated again and again. We prove that 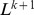 many searches are sufficient in order to find the target in polynomial time with high probability (see Figure 4 and Section 10 in Text S1). The upper bound, 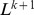, holds even for neutral drift (without selection). Note that in this case, the expected discovery time for any single search is still exponential. Therefore, most of the 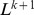 searches do not succeed in polynomial time; however, with high probability one of the searches succeeds in polynomial time. There are two key aspects to the “regeneration process”: (a) the starting sequence is only a small number of steps away from the target; and (b) the starting sequence can be generated repeatedly. This process enables evolution to overcome the exponential barrier. The upper bound, 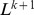, may possibly be further reduced, if selection and/or recombination are included.

## Discussion

The regeneration process formalizes the role of several existing ideas. First, it ties in with the proposal that gene duplications and genome rearrangements are major events leading to the emergence of new genes [43]. Second, evolution can be seen as a tinkerer playing around with small modifications of existing sequences rather than creating entirely new ones [44]. Third, the process is related to Gillespie's suggestion [29] that the starting sequence for an evolutionary search must have high fitness. In our theory, proximity in fitness value is replaced by proximity in sequence space. However, our results show that proximity alone is insufficient to break the exponential barrier, and only when combined with the process of regeneration it yields polynomial discovery time with high probability. Our process can also explain the emergence of orphan genes arising from non-coding regions [45]. Section 12 of the Text S1 discusses the connection of our approach to existing results.

There is one other scenario that must be mentioned. It is possible that certain biological functions are hyper-abundant in sequence space [21] and that a process generating a large number of random sequences will find the function with high probability. For example, Bartel & Szostak [46] isolated a new ribozyme from a pool of about 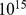 random sequences of length 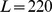. While such a process is conceivable for small effective sequence length, it cannot represent a general solution for large 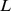.

Our theory has clear empirical implications. The regeneration process can be tested in systems of in vitro evolution [47]. A starting sequence can be generated by introducing 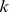 point mutations in a known protein encoding sequence of length 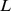. If these point mutations destroy the function of the protein, then the expected discovery time of any one attempt to find the original sequence should be exponential in 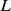. But only polynomially many searches in 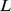 are required to find the target with high probability in polynomially many steps. The same setup can be used to explore whether the biological function can be found elsewhere in sequence space: the evolutionary trajectory beginning with the starting sequence could discover new solutions. Our theory also highlights how important it is to explore the distribution of biological functions in sequence space both for RNA [20], [21], [35], [46] and in the protein universe [48].

In summary, we have developed a theory that allows us to estimate time scales of evolutionary trajectories. We have shown that various natural processes of evolution take exponential time as function of the sequence length, 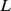. In some cases we have established strong dichotomy results for precise boundary conditions. We have proposed a mechanism that allows evolution in polynomial time scales. Some interesting directions of future work are as follows: (1) Consider various forms of rugged fitness landscapes and study more refined approximations as compared to the ones we consider; and then estimate the expected discovery time for the refined approximations. (2) While in this paper we characterize the difference between exponential and polynomial for the expected discovery time, more refined analysis (such as efficiency for polynomial time, like cubic vs quadratic time) for specific fitness landscapes using mechanisms like recombination is another interesting problem.

## Materials and Methods

Our results are based on a mathematical analysis of the underlying stochastic processes. For Markov chains on the one-dimensional grid, we describe recurrence relations for the expected hitting time and present lower and upper bounds on the expected hitting time using combinatorial analysis (see Text S1 for details). We now present the basic intuitive arguments of the main results.

## 

### Markov chain on the one-dimensional grid

For a single broad peak, due to symmetry we can interpret the evolutionary random walk as a Markov chain on the one-dimensional grid. A sequence of type 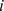 is 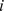 steps away from the target, where 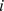 is the Hamming distance between this sequence and the target. The probability that a type 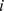 sequence mutates to a type 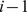 sequence is given by 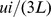. The stochastic process of the evolutionary random walk is a Markov chain on the one-dimensional grid 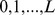.

### The basic recurrence relation

Consider a Markov chain on the one-dimensional grid, and let 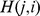 denote the expected hitting time from 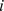 to 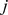. The general recurrence relation for the expected hitting time is as follows:
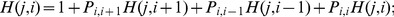
(1)for 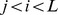, with boundary condition 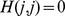. The interpretation is as follows. Given the current state 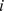, if 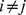, at least one transition will be made to a neighboring state 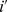, with probability 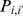, from which the hitting time is 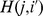.

### Intuition behind Theorem 1

Theorem 1 is derived by obtaining precise bounds for the recurrence relation of the hitting time (Equation 1). Consider that 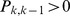 for all 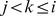 (i.e., progress towards state 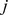 is always possible), as otherwise 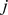 is never reached from 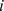. We show (see Lemma 2 in the Text S1) that we can write 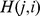 as a sum, 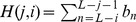, where 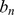 is the sequence defined as:
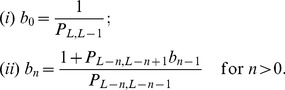
(2)


The basic intuition obtained from Equation 2 is as follows: (i) If 
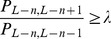
, for some constant 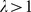, then the sequence 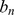 grows at least as fast as a geometric series with factor 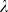. (ii) On the other hand, if 
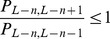
 and 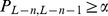 for some constant 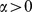, then the sequence 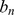 grows at most as fast as an arithmetic series with difference 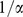. From the above case analysis the result for Theorem 1 is obtained as follows: If 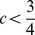, then for all 
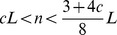
, we have 
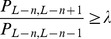
 for some 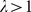, and hence the sequence 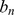 grows geometrically for a linear length in 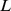. Then, 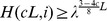 for all states 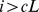 (i.e., for all sequences outside of the target set). This corresponds to case 1 of Theorem 1. On the other hand, if 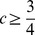, then it is 
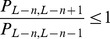
, and case 2 of Theorem 1 is derived (for details see Corollary 2 in Text S1).

### Intuition behind Theorem 2

The basic intuition for the result is as follows: consider a single search for which the expected hitting time is exponential. Then for the single search the probability to succeed in polynomially many steps is negligible (as otherwise the expectation would not have been exponential). In case of independent searches, the independence ensures that the probability that all searches fail is the product of the probabilities that every single search fails. Using the above arguments we establish Theorem 2 (for details see Section 8 in Text S1).

### Intuition behind Theorem 3

For this result, it is first convenient to view the evolutionary walk taking place in the sequence space of all sequences of length 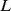, under no selection. Each sequence has 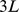 neighbors, and considering that a point mutation happens, the transition probability to each of them is 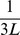. The underlying Markov chain due to symmetry has fast mixing time, i.e., the number of steps to converge to the stationary distribution (the mixing time) is 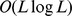. Again by symmetry the stationary distribution is the uniform distribution. If 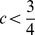, then from Theorem 1 we obtain that the expected time to reach a single broad peak is exponential. By union bound, if 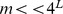, the probability to reach any of the 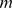 broad peaks within 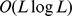 steps is negligible. Since after the first 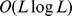 steps the Markov chain converges to the stationary distribution, then each step of the process can be interpreted as selection of sequences uniformly at random among all sequences. Using Hoeffding's inequality, we show that with high probability, in expectation 
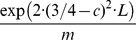
 such steps are required before a sequence is found that belongs to the target set. Thus we obtain the result of Theorem 3 (for details see Section 9 in Text S1).

### Remark about techniques

An important aspect of our work is that we establish our results using elementary techniques for analysis of Markov chains. The use of more advanced mathematical machinery, such as martingales [49] or drift analysis [50], [51], can possibly be used to derive more refined results. While in this work our goal is to distinguish between exponential and polynomial time, whether the techniques from [49]–[51] can lead to a more refined characterization within polynomial time is an interesting direction for future work.

## Supporting Information

Text S1 Detailed proofs for “The Time Scale of Evolutionary Innovation.”
(PDF)
