# Supplementary material for: The Time Scale of Evolutionary Innovation
Source: PLoS Comput Biol. 2014 Sep 11;10(9):e1003818. doi: 10.1371/journal.pcbi.1003818 (PMC4161296; doi:10.1371/journal.pcbi.1003818)
Supplement: Text S1 — Detailed proofs for “The Time Scale of Evolutionary Innovation.” (PDF) [file pcbi.1003818.s001.pdf]

Text S1: Detailed proofs for "The time scale of evolutionary innovation"

Krishnendu Chatterjee   Andreas Pavlogiannis   Ben Adlam   Martin A. Nowak

1. Overview and Organization

We will present detailed proofs of all our results. In this section we present an overview of the proof structure and the organization of our results.

1. In Section 2 we present relevant lower and upper bounds on hitting time for Markov chains on an one-dimensional grid. The results of this section are technical and the basis for the results of the following sections. However, a reader does not need to understand the technical proofs of this section for the following sections. We will only use the results of Lemma S3 and Lemma S4 (and their consequence Corollary S1); and Lemma S5 (and its implication) in the following subsections. We present the results in the most general form for Markov chains, and they might possibly be used in other contexts as well; and then present simple applications of the general results of Markov chains for the discovery time of evolutionary processes.
2. In Section 3 we introduce evolutionary processes and for simplicity we introduce them for evolutionary adaptation of bit strings. Also for mathematically elegant proofs we first introduce the Fermi evolutionary process in this section, and later consider the Moran process.
3. In Section 4 we present our results for the Fermi evolutionary process with neutral fitness landscapes and a broad peak of targets.
4. In Section 5 we present our results for constant selection in the Fermi evolutionary process with a broad peak of targets.
5. In Section 6 we show how the results of Section 4 and Section 5 imply all the desired results for the Moran evolutionary process.
6. In Section 7 we show how the results of Section 4, Section 5, and Section 6, extend from bit strings to strings over alphabet of any size (and obtain the results for four letter alphabet as a special case).
7. In Section 8 we present the results for multiple independent searches; and in Section 9 we discuss some cases of distributed targets.
8. In Section 10 we discuss the results for a mechanism to enable evolution to work in polynomial time. Finally, in Section 11 we present details of some numerical calculations used in the main article.
9. In Section 12 we discuss and compare our results with relevant related work, and end with additional simulation results in Section 13.

While for simplicity in the main article we only present our results for neutral fitness landscapes, Sections 5, 6 and 7 deal with the more general case of selection acting on multiplicative fitness landscapes.

2. Bounds on hitting times of Markov chains on a line

In this section we will present our basic lower and upper bounds on hitting times of Markov chains on a line. The results of this section will be used repeatedly in the later sections to provide lower and upper bounds on the discovery time for several evolutionary processes. We start with the definition of Markov chains, and then define the special case of Markov chains on a line.

Definition S1 (Markov chains)**.** *A finite-state Markov chain $MC_L = (S, \delta)$ consists of a finite set S of states, with $S = \{0, 1, \dots L\}$ (i.e., the set of states is a finite subset of the natural numbers starting from 0), and a stochastic transition matrix δ that specifies the transition probabilities, i.e., $\delta(i, j)$ denotes the probability of transition from state i to state j (in other words, for all $0 \leq i, j \leq L$ we have $0 \leq \delta(i, j) \leq 1$ and for all $0 \leq i \leq L$ we have $\sum_{j=0}^{L} \delta(i, j) = 1$).*

We now introduce Markov chains on a line. Intuitively a Markov chain on a line is defined as a special case of Markov chains, for which in every state, the allowed transitions are either self-loops, or to the left, or to the right. The formal definition is as follows.

Definition S2 (Markov chains on a line)**.** *A* Markov chain on a line*, denoted as M_L, is a finite-state Markov chain (S, δ) where $S = \{0, 1, \dots L\}$ and for all $0 \leq i, j \leq L$, if $\delta(i, j) > 0$, then $|i - j| \leq 1$, i.e., the transitions allowed are only self-loops, to the left, and to the right (see Supplementary Figure 1).*

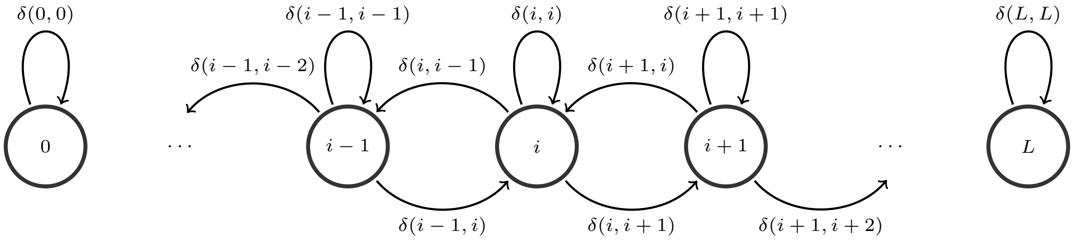

Supplementary Figure 1: Markov chain on a line. Pictorial illustration of Markov chain on a line.

We now define the notion of hitting times for Markov chains on a line.

Definition S3 (Hitting time)**.** *Given a Markov chain on a line M_L, and two states n_1 and n_2 (i.e., $0 \leq n_1, n_2 \leq L$), we denote by $H(n_1, n_2)$ the expected hitting time from the starting state n_2 to the target state n_1, i.e., the expected number of transitions required to reach the target state n_1 starting from the state n_2.*

The recurrence relation for hitting time. Given a Markov chain on a line $M_L = (S, \delta)$, and a state n_1 (i.e., $0 \leq n_1 \leq L$), the following recurrence relation holds:

1. $H(n_1, n_1) = 0$,
2. $H(n_1, i) = 1 + \delta(i, i+1) \cdot H(n_1, i+1) + \delta(i, i-1) \cdot H(n_1, i-1) + \delta(i, i) \cdot H(n_1, i)$, for all $n_1 < i < L$, and
3. $H(n_1, L) = 1 + \delta(L, L-1) \cdot H(n_1, L-1) + \delta(L, L) \cdot H(L, L)$.

The argument is as follows: (a) Case 1 is trivial. (b) For case 2, since $i \neq n_1$, at least one transition needs to be taken to a neighbor state j of i, from which the hitting time is $H(n_1, j)$. With probability $\delta(i, i+1)$ the neighbor j is state $i+1$, while with probability $\delta(i, i-1)$ the neighbor j is state $i-1$. On the other hand, with probability $\delta(i, i)$ the self-loop transition is taken, and the expected hitting time remains the same.

(c) Case 3 is a degenerate version of case 2, where the only possible transitions from the state L are either to the state $L-1$, which is taken with probability $\delta(L, L-1)$, or the self-loop, which is taken with probability $\delta(L, L)$. Also note that in Case 3 we have $\delta(L, L-1) = 1 - \delta(L, L)$. In the following lemma we show that using the recurrence relation, the hitting time can be expressed as the sum of a sequence of numbers.

Lemma S1. *Consider a Markov chain on a line M_L, with a target state n_1, such that for all $n_1 < i \leq L$ we have $\delta(i, i-1) > 0$. For all $n_1 < i \leq L$ we have that $H(n_1, i) = \sum_{j=L-i}^{L-n_1-1} b_j$, where b_j is the sequence defined as:*

$$(1)\ b_0 = \frac{1}{\delta(L, L-1)}; \qquad (2)\ b_j = \frac{1 + \delta(L-j, L-j+1) \cdot b_{j-1}}{\delta(L-j, L-j-1)} \quad \textit{for } j > 0.$$

Proof. We consider the recurrence relation for the hitting time and first show that for all $0 \leq i < L - n_1$ we can write $H(n_1, L-i)$ as

$$H(n_1, L-i) = b_i + H(n_1, L-i-1)$$

for the desired sequence b_i.

(Base case). For $i = 0$ we have

$$\begin{aligned} H(n_1, L) &= 1 + \delta(L, L-1) \cdot H(n_1, L-1) + \delta(L, L) \cdot H(n_1, L) \\ &= 1 + \delta(L, L-1) \cdot H(n_1, L-1) + (1 - \delta(L, L-1)) \cdot H(n_1, L) \\ &= \frac{1}{\delta(L, L-1)} + H(n_1, L-1), \end{aligned}$$

thus the statement holds with $b_0 = \frac{1}{\delta(L, L-1)}$.

(Inductive case). Assume that the statement holds for some $i-1$ (inductive hypothesis) and we will show that it also holds for i. Let $y = \delta(L-i, L-i+1)$ and $x = \delta(L-i, L-i-1)$. We establish the following equality:

$$\begin{aligned} H(n_1, L-i) &= 1 + y \cdot H(n_1, L-i+1) + x \cdot H(n_1, L-i-1) + (1-x-y) \cdot H(n_1, L-i) \\ &= 1 + y \cdot \big(b_{i-1} + H(n_1, L-i)\big) + x \cdot H(n_1, L-i-1) + (1-x-y) \cdot H(n_1, L-i) \\ &= \frac{1 + y \cdot b_{i-1}}{x} + H(n_1, L-i-1). \end{aligned}$$

The first equality follows from the recurrence relation (case 2) by substituting i with $L-i$; the second equality follows by substitution and the inductive hypothesis; the third equality is simple re-writing, since $x \neq 0$. Thus we have $H(n_1, L-i) = b_i + H(n_1, L-i-1)$, where

$$(1)\ b_0 = \frac{1}{\delta(L, L-1)}; \qquad (2)\ b_j = \frac{1 + \delta(L-j, L-j+1) \cdot b_{j-1}}{\delta(L-j, L-j-1)} \quad \text{for } j > 0.$$

Hence we have $H(n_1, L-i) = \sum_{j=i}^{L-n_1-1} b_j$ and by substituting back $i \leftarrow L-i$ we obtain $H(n_1, i) = \sum_{j=L-i}^{L-n_1-1} b_j$. The desired result follows. □

Definition S4. *For positive real-valued constants A and B, we define the sequence $a_i(A, B)$ as follows:*

$$(1)\ a_0(A, B) = \frac{1}{B} \qquad (2)\ a_i(A, B) = \frac{1 + A \cdot a_{i-1}(A, B)}{B} \quad \textit{for } i > 0.$$

Lemma S2. *For positive real-valued constants A and B, the following assertions hold for the sequence $a_i(A, B)$:*

- *If $A > B$ and $B \leq 1$, then $a_i(A,B) \geq \left(\frac{A}{B}\right)^i$, with $\frac{A}{B} > 1$.*
- *If $A \leq B$, then $a_i(A,B) = O(\frac{i}{B})$.*

Proof. The result is as follows:

- Case $A > B$: Then we have

$$a_i(A,B) = \frac{1 + A \cdot a_{i-1}(A,B)}{B} > \frac{A}{B} \cdot a_{i-1}(A,B) \geq \left(\frac{A}{B}\right)^i \cdot a_0(A,B) = \left(\frac{A}{B}\right)^i \cdot \frac{1}{B} \geq \left(\frac{A}{B}\right)^i$$

(by just ignoring the term 1 in the numerator and since $B \leq 1$).

- Case $A \leq B$: Then $\frac{A}{B} \leq 1$ and $a_i(A,B) = \frac{1+A \cdot a_{i-1}(A,B)}{B} \leq \frac{1}{B} + a_{i-1}(A,B) \leq \frac{i}{B} + \frac{1}{B} = O(\frac{i}{B})$.

The desired result follows. □

Exponential lower bound. We will use the following standard convention in this paper: a function $t(L)$ is lower bounded by an exponential function, if there exist constants $c > 1$, $\ell > 0$ and $L_0 \in \mathbb{N}$ such that for all $L \geq L_0$ we have $t(L) \geq c^{\ell \cdot L} = 2^{c^* \cdot \ell \cdot L}$, where $c^* = \log c > 0$, i.e., it is lower bounded by a linear function in the exponent.

Exponential lower bound on hitting times for Markov chains on a line. In the following lemma we will show an exponential lower bound on the hitting time. We consider a Markov chain on a line M_L, such that there exist two states x and $y = x + k$, for $k > 0$, such that in the whole contiguous segment between x and y the ratio of the probability to drift towards the right as compared to the left is at least $1 + A$, for a constant $A > 0$ (strictly bounded away from 1). Then the expected hitting time from any starting point right of x to a target to the left of x is at least $(1 + A)^{k-1}$.

Lemma S3 (Lower bound). *Consider a Markov chain on a line M_L. If there exist two states $x, y \leq L$ with $y = x + k$, for $k > 0$, and a constant $A > 0$ such that for all $x < i < y$ we have $\frac{\delta(i,i+1)}{\delta(i,i-1)} \geq 1 + A$, then for all $n_1, n_2 \leq L$ such that $n_1 \leq x < n_2$ we have $H(n_1, n_2) \geq (1 + A)^{k-1}$.*

Proof. From Lemma S1 we have that $H(n_1, n_2) = \sum_{j=L-n_2}^{L-n_1-1} b_j$:

$$H(n_1, n_2) = \sum_{j=L-n_2}^{L-n_1-1} b_j \geq \sum_{j=L-(x+1)}^{L-(x+1)} b_j = b_{L-x-1}.$$

We have $\frac{\delta(i,i+1)}{\delta(i,i-1)} \geq 1 + A$ by the given condition of the lemma. We show by induction that for all j between $L - y$ and $L - x - 1$ (i.e., $L - y \leq j \leq L - x - 1$) we have $b_j \geq a_{j-L+y}(1 + A, 1)$.

1. *(Base case).* We have $b_{L-y} \geq 1 = a_0(1 + A, 1)$, since b_j is non-decreasing and $b_0 = \frac{1}{\delta(L,L-1)} \geq 1$.
2. *(Inductive case).* By inductive hypothesis on $j - 1$ we have $b_{j-1} \geq a_{j-1-L+y}(1 + A, 1)$, and then we have

$$b_j = \frac{1 + \delta(L-j, L-j+1) \cdot b_{j-1}}{\delta(L-j, L-j-1)} \geq 1 + (1 + A) \cdot a_{j-1-L+y}(1 + A, 1) = a_{j-L+y}(1 + A, 1)$$

since $\frac{\delta(L-j,L-j+1)}{\delta(L-j,L-j-1)} \geq 1 + A$ and $\delta(L-j, L-j-1) \leq 1$. Thus we have $b_j \geq a_{j-L+y}(1 + A, 1)$.

Thus for all $L - y \leq j \leq L - x - 1$ we have $b_j \geq a_{j-L+y}(1 + A, 1)$. Hence $H(n_1, n_2) \geq b_{L-x-1} \geq a_{y-x-1}(1 + A, 1) = a_{k-1}(1 + A, 1) \geq (1 + A)^{k-1}$ (from Lemma S2, since $1 + A > 1$). □

Lemma S4 (Upper bound)**.** *Given a Markov chain on a line M_L and $0 \leq n_1 < n_2 \leq L$, if for all $n_1 < i < L$ we have $\delta(i, i-1) \geq \delta(i, i+1)$, then $H(n_1, n_2) = O(\frac{L^2}{B^*})$, where $B^* = \min_{n_1 < i \leq L}(1 - \delta(i, i))$.*

Proof. From Lemma S1 we have that $H(n_1, n_2) = \sum_{j=L-n_2}^{L-n_1-1} b_j$. Let $B = \min_{n_1 < i \leq L} \delta(i, i-1)$. We show by induction that for all $0 \leq j \leq L - n_1 - 1$ we have $b_j \leq a_j(1, B)$.

1. *(Base case).* We have $b_0 = \frac{1}{\delta(L,L-1)} \leq \frac{1}{B} = a_0(A, B)$ (because of our choice of B we have $B \leq \delta(L, L-1)$).

2. *(Inductive case).* By inductive hypothesis on $j-1$ we have $b_{j-1} \leq a_{j-1}(1, B)$. Then

$$b_j = \frac{1 + \delta(L-j, L-j+1) \cdot b_{j-1}}{\delta(L-j, L-j-1)} \leq \frac{1}{\delta(L-j, L-j-1)} + \frac{\delta(L-j, L-j+1) \cdot a_{j-1}(1, B)}{\delta(L-j, L-j-1)}$$
$$\leq \frac{1}{B} + a_{j-1}(1, B) \leq \frac{1 + a_{j-1}(1, B)}{B} = a_j(1, B).$$

since $\frac{1}{\delta(L-j,L-j-1)} \leq \frac{1}{B}$, $\frac{\delta(L-j,L-j+1)}{\delta(L-j,L-j-1)} \leq 1$, and $\frac{1}{B} \geq 1$. Thus $b_j \leq a_j(1, B)$.

It follows that for all $L - n_2 \leq j \leq L - n_1 - 1$ we have $b_j \leq a_j(1, B)$ and thus $b_j = O(\frac{j}{B})$ from Lemma S2. Then $H(n_1, n_2) = \sum_{j=L-n_2}^{L-n_1-1} b_j = O\left((n_2 - n_1) \cdot (L - n_1 - 1) \cdot \frac{1}{B}\right) = O(\frac{L^2}{B})$. Let $j = \arg\min_{n_1 < i \leq L} \delta(i, i-1)$. We have

$$B = \delta(j, j-1) \geq \frac{1}{2} \cdot (\delta(j, j-1) + \delta(j, j+1)) = \frac{1}{2} \cdot (1 - \delta(j, j)) \geq \frac{1}{2} \cdot B^*$$

because $\delta(j, j-1) \geq \delta(j, j+1)$ and $1 - \delta(j, j) \geq B^*$. We conclude that $H(n_1, n_2) = O(\frac{L^2}{B^*})$. □

Markov chains on a line without self-loops. A special case of the above lemma is obtained for Markov chains on a line with no self-loops in states other than state 0, i.e., for all $0 < i \leq L$ we have $1 - \delta(i, i) = 1 = B^*$. We consider a Markov chain on a line without self-loops M_L, such that there exist two states x and $y = x + k$, for $k > 0$, such that in the whole contiguous segment between x and y the probability to drift towards the right is at least a constant $A > \frac{1}{2}$ (strictly bounded away from $\frac{1}{2}$). We also assume $A < 1$, since otherwise transitions to the left are never taken. Then the expected hitting time from any starting point right of x to a target to the left of x is at least c_A^{k-1}, where $c_A = \frac{A}{1-A} > 1$ (see Supplementary Figure 2).

Corollary S1. *Given a Markov chain on a line M_L such that for all $0 < i \leq L$ we have $\delta(i, i) = 0$, the following assertions hold:*

1. Lower bound: *If there exist two states $x, y \leq L$ with $y = x + k$, for $k > 0$, and a constant $A > \frac{1}{2}$ such that for all $x \leq i < y$ we have $\delta(i, i+1) \geq A > \frac{1}{2}$, then for all $n_1, n_2 \leq L$ such that $n_1 \leq x < n_2$ we have $H(n_1, n_2) \geq c_A^{k-1}$ for $c_A = \frac{A}{1-A} > 1$.*

2. Upper bound: *For $0 \leq n_1 < n_2 \leq L$, if for all $n_1 < i < L$ we have $\delta(i, i-1) \geq \frac{1}{2}$, then $H(n_1, n_2) = O(L^2)$.*

Proof. Since $\delta(i, i) = 0$, we have that $\delta(i, i+1) \geq A$ implies that $\frac{\delta(i,i+1)}{\delta(i,i-1)} \geq \frac{A}{1-A}$, and then the first item is an easy consequence of Lemma S3. For item (2), we have $\delta(i, i-1) \geq \frac{1}{2}$ implies $\delta(i, i-1) \geq \delta(i, i+1)$ and hence the result follows from Lemma S4 with $B^* = 1$ since $\delta(j, j) = 0$ for all $n_1 < j < L$. □

Unloop variant of Markov chains on a line. We will now show how given a Markov chain on a line with self-loops we can create a variant without self-loops and establish a relation on the hitting time of the original Markov chain and its variant without self-loops.

Definition S5 (Unloop variant of Markov chain on a line)**.** *Given a Markov chain on a line $M_L = (S, \delta)$, we call its* unloop variant *a Markov chain on a line $\overline{M}_L = (S, \overline{\delta})$, with the following properties:*

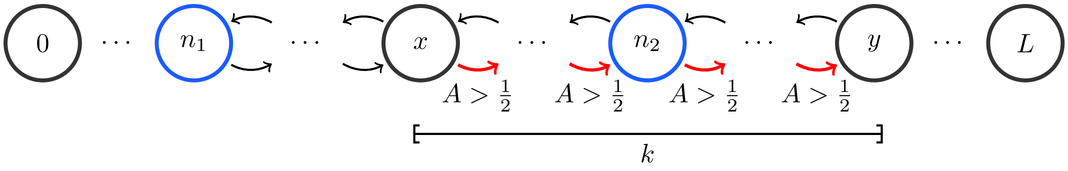

(a)

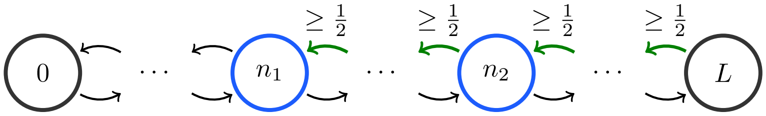

(b)

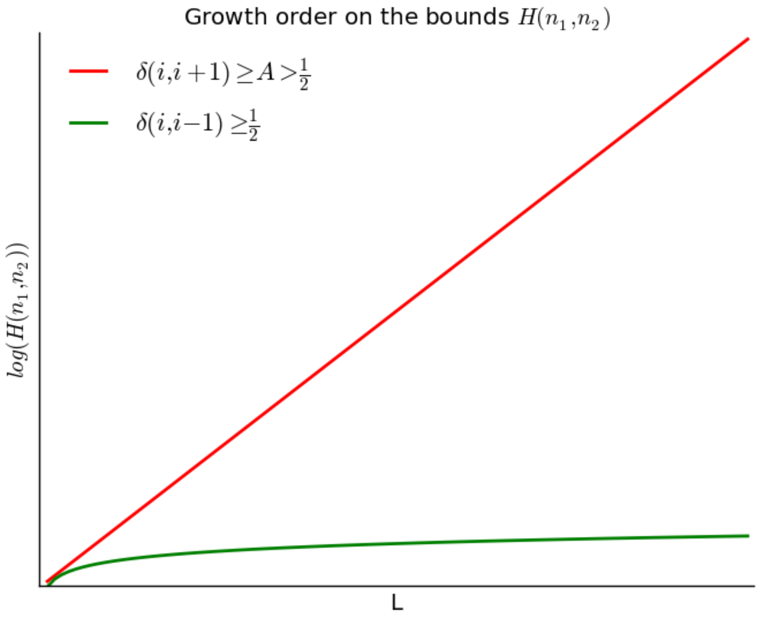

(c)

Supplementary Figure 2: Lower and upper bound on hitting times for Markov chain on a line. Figure (a) shows a Markov chain on a line without self-loops, where for a length k between x and y the transition probabilities to the right are at least a constant $A > \frac{1}{2}$, and then the hitting time from any starting point n_2 to the right of x to a target n_1 to the left of x is at least exponential in the length k; figure (b) shows a Markov chain on a line without self-loops where all the transition probabilities to the left upto the target n_1 are at least $\frac{1}{2}$, and then the hitting time for any start point to the right of the target n_1 to the target is at most $O(L^2)$; the graph (c) shows the exponential lower bound (red) and polynomial upper bound (green) on the hitting times $H(n_1, n_2)$ in the log-scale.

- $\overline{\delta}(0,1) = 1$;
- *For all* $0 < i < L$, *we have* $\overline{\delta}(i,i-1) = \frac{\delta(i,i-1)}{\delta(i,i-1)+\delta(i,i+1)}$ *and* $\overline{\delta}(i,i+1) = \frac{\delta(i,i+1)}{\delta(i,i-1)+\delta(i,i+1)}$, *i.e., the probabilities of transitions to right and left are normalized so that they sum to 1; and*
- $\overline{\delta}(L,L-1) = 1$.

We now show the following: (1) the hitting time of the original Markov chain on a line M_L is always at least the hitting time of the unloop variant; and (2) the hitting time of the original Markov chain is at most z^* times the hitting time of the unloop variant, where z^* is the maximum of the inverse of the 1 minus the self-loop transition probabilities.

Lemma S5. *Consider a Markov chain on a line* $M_L = (S,\delta)$ *and its unloop variant* $\overline{M}_L = (S,\overline{\delta})$. *Let* $0 < n_1, n_2 \leq L$ *and* $n_1 < n_2$, *and let* $H(n_1,n_2)$ *denote the hitting time to state* n_1 *from state* n_2 *in* M_L, *and* $\overline{H}(n_1,n_2)$ *denote the corresponding hitting time in* $\overline{M}_L$. *The following assertions hold:*

(i) $\overline{H}(n_1,n_2) \leq H(n_1,n_2)$.

(ii) $H(n_1,n_2) \leq z^* \cdot \overline{H}(n_1,n_2)$, *where* $z^* = \max_{0<i\leq L} \frac{1}{1-\delta(i,i)}$.

Proof. From Lemma S1 we have that for all $0 < i \leq L$, we can write $H(n_1,i) = \sum_{j=L-i}^{L-n_1-1} b_j$ and $\overline{H}(n_1,i) = \sum_{j=L-i}^{L-n_1-1} \overline{b}_j$ where b_j and $\overline{b}_j$ are the sequences defined as:

$$(1)\ b_0 = \frac{1}{\delta(L,L-1)}; \qquad (2)\ b_j = \frac{1+\delta(L-j,L-j+1)\cdot b_{j-1}}{\delta(L-j,L-j-1)} \quad \text{for } j>0.$$

and

$$(1)\ \overline{b}_0 = 1; \qquad (2)\ \overline{b}_j = \frac{1+\overline{\delta}(L-j,L-j+1)\cdot \overline{b}_{j-1}}{\overline{\delta}(L-j,L-j-1)} \quad \text{for } j>0.$$

(i) We prove inductively that for all $0 < j < L-1$, we have $\overline{b}_j \leq b_j$.

1. *(Base case).* $\overline{b}_0 = 1 \leq \frac{1}{\delta(L,L-1)} = b_0$.
2. *(Inductive Step).* The inductive hypothesis guarantees that $\overline{b}_{j-1} \leq b_{j-1}$. Observe that $\frac{\overline{\delta}(L-j,L-j+1)}{\overline{\delta}(L-j,L-j-1)} = \frac{\delta(L-j,L-j+1)}{\delta(L-j,L-j-1)} = R$. Then

$$\begin{aligned}\overline{b}_j &= \frac{1+\overline{\delta}(L-j,L-j+1)\cdot \overline{b}_{j-1}}{\overline{\delta}(L-j,L-j-1)} = \frac{1}{\overline{\delta}(L-j,L-j-1)} + R\cdot \overline{b}_{j-1} \\ &\leq \frac{1}{\delta(L-j,L-j-1)} + R\cdot b_{j-1} = \frac{1+\delta(L-j,L-j+1)\cdot b_{j-1}}{\delta(L-j,L-j-1)} = b_j\end{aligned}$$

because of the inductive hypothesis and $\overline{\delta}(L-j,L-j-1) \geq \delta(L-j,L-j-1)$.

Thus for all such j, we have $\overline{b}_j \leq b_j$, and $\overline{H}(n_1,n_2) = \sum_{j=L-n_2}^{L-n_1-1} \overline{b}_j \leq \sum_{j=L-n_2}^{L-n_1-1} b_j = H(n_1,n_2)$.

(ii) We prove inductively that for all $0 < j < L-1$, we have $b_j \leq z^* \cdot \overline{b}_j$.

1. *(Base case).* $b_0 = \frac{1}{\delta(L,L-1)} = \frac{1}{1-\delta(L,L)} \leq z^* = z^* \cdot \overline{b}_0$.
2. *(Inductive Step).* The inductive hypothesis guarantees that $b_{j-1} \leq z^* \cdot \overline{b}_{j-1}$. Observe that $\frac{\overline{\delta}(L-j,L-j+1)}{\overline{\delta}(L-j,L-j-1)} = \frac{\delta(L-j,L-j+1)}{\delta(L-j,L-j-1)} = R$. Moreover, let $x = \delta(L-j,L-j-1)$ and $y = \delta(L-j,L-j+1)$, and then we have:

$$z^* \geq \frac{1}{1-\delta(L-j,L-j)} \implies z^* \geq \frac{1}{x+y} \implies z^* \cdot (x+y) \geq 1 \implies z^* \cdot \frac{x+y}{x} \geq \frac{1}{x}.$$

Thus

$$b_j = \frac{1+y\cdot b_{j-1}}{x} = \frac{1}{x} + R\cdot b_{j-1} \leq z^* \cdot \frac{x+y}{x} + R\cdot z^* \cdot \overline{b}_{j-1} = z^* \cdot \frac{1+\overline{\delta}(L-j,L-j+1)\cdot \overline{b}_{j-1}}{\overline{\delta}(L-j,L-j-1)} = z^* \cdot \overline{b}_j$$

since $\frac{x+y}{x} = \frac{1}{\overline{\delta}(L-j,L-j-1)}$.

Thus for all $0 < j < L-1$, we have $b_j \leq z^* \cdot \overline{b}_j$, and hence $H(n_1,n_2) = \sum_{j=L-n_2}^{L-n_1-1} b_j \leq \sum_{j=L-n_2}^{L-n_1-1} z^* \cdot \overline{b}_j = z^* \cdot \overline{H}(n_1,n_2)$.

This completes the proof. □

Implication of Lemma S5. The main implication of Lemma S5 is as follows: any lower bound on the hitting time on the unloop variant is a lower bound on the hitting time of the original Markov chain; and an upper bound on the hitting time on the unloop variant multiplied by z^* gives an upper bound on the hitting time of the original Markov chain.

3. Evolutionary Process

In this section we consider a simple model of evolutionary process, where organisms/genotypes are represented as strings of length L, and view evolution as a discrete time process. For simplicity, we will first consider the case of bit strings and present all our results with bit strings because all the key proof ideas are illustrated there. We will then generalize our results to strings for any alphabet size in Section 7. For a bit string s, at any time point a random mutation can appear with probability u, which will invert a single bit of the string s. Such mutations can be viewed as transitions between genotypes which form a random walk in the L-dimensional genotypic space of all 2^L strings.

Notations. For $L \in \mathbb{N}$, we denote by $B(L)$ the set of all L-bit strings. Given a string $s \in B(L)$, the neighborhood $\mathsf{Nh}(s)$ of s is the set of strings that differ from s by only one bit, i.e., $\mathsf{Nh}(s) = \{s' \in B(L) : s, s'$ differ in exactly one position$\}$. In order to model natural selection, we will consider a constant *selection intensity* $\beta \in \mathbb{R}$ and each string s will be associated with a fitness according to a *fitness function* $f(s) \in \mathbb{R}$. The selection intensity and the fitness function will determine the transition probabilities between s and its neighbors.

Transition probability between strings. Given a string s and $s' \in \mathsf{Nh}(s)$, the transition probability $\Delta(s,s')$ from s to s' depends (i) on the fitness of s and the fitness of the neighbors in $\mathsf{Nh}(s)$, and (ii) the selection intensity. For all $s'' \in \mathsf{Nh}(s)$, let $df(s,s'') = (f(s'') - f(s))$ denote the difference in fitness of s and s'', and let $g(s,s'') = \frac{1}{1+e^{-\beta\cdot df(s,s'')}}$. Then the transition probability is defined as follows:

$$\Delta(s,s') = u \cdot \frac{g(s,s')}{\sum_{s''\in \mathsf{Nh}(s)} g(s,s'')} \tag{1}$$

The intuitive description of the transition probability (which is refered as Fermi process) is as follows: the term u represents the probability of a mutation occurring in s, while the choice of the neighbor s' is based on a normalized weighted sum, with each sigmoid term $\frac{1}{1+e^{-\beta\cdot df(s,s')}}$ being determined by the fitness difference between s, s' and the selection intensity. The selection intensity acts like the temperature function. The high values of the selection intensity will favor those transitions to neighbors that have higher fitness, while

setting $\beta = 0$ turns all the possible transitions of equal probability and independent of the fitness landscape (we refer to this case as *neutral* selection).

Discovery time. Given a string space $B(L)$, a fitness function f and a selection intensity β, for two strings s_1, $s_2 \in B(L)$, we denote by $T(s_1, s_2, f, \beta)$ the expected discovery time of the target string s_1 from the starting string s_2, i.e., the average number of steps necessary to transform s_2 to s_1 under the fitness landscape f and selection intensity β. Given a start string s_2 and a target set U of strings we denote by $T(U, s_2, f, \beta)$ the expected discovery time of the target set U starting from the string s_2, i.e., the average number of steps necessary to transform s_2 to some string in U. In the following section we will present several lower and upper bounds on the discovery times depending on the fitness function and selection intensity.

Moran evolutionary process. The evolutionary process we described is the Fermi process where the transition probabilities are chosen according to the Fermi function and the fitness difference. We will first present lower and upper bounds for the Fermi evolutionary process for mathematically elegant proofs, and then argue how the bounds are easily transferred to the Moran evolutionary process.

4. Neutral Selection

In this section we consider the case of neutral selection, and hence the transition probabilities are independent of the fitness function. Since $\beta = 0$ for all strings s, the transition probability equation (Eqn 1) simplifies to $\Delta(s, s') = \frac{u}{L}$ for all $s' \in \mathsf{Nh}(s)$. We will present an exponential lower bound on the discovery time of a set of targets concentrated around the sequence $\vec{0}$, and we will refer to this case as *broad peak*. For a constant $0 < c < 1$, let U_c^L denote the set of all strings such that at most cL bits are ones (i.e., at least $(1-c) \cdot L$ bits are zeros). In other words, U_c^L is the set of strings that have Hamming distance at most cL to $\vec{0}$. We consider the set U_c^L as the target set. Because there is neutral selection the fitness landscape is immaterial, and for the sequel of this section we will drop the last two arguments of $T(\cdot, \cdot, f, \beta)$ since $\beta = 0$ and the discovery time is independent of f.

We model the evolutionary process as a Markov chain on a line, $M_{L,0} = (S, \delta_0)$ (0 for neutral), which is obtained as follows: by symmetry, all strings that have exactly i-ones and $(L-i)$-zeros form an equivalence class, which is represented as state i of the Markov chain. The transition probabilities from state i are as follows: (i) for $0 < i < L$ we have $\delta_0(i, i-1) = \frac{u \cdot i}{L}$ and $\delta_0(i, i+1) = \frac{u \cdot (L-i)}{L}$; (ii) $\delta_0(0, 1) = u$; and (iii) $\delta_0(L, L-1) = u$. Then we have the following equivalence: for a string s in $B(L) \setminus U_c^L$ the discovery time $T(U_c^L, s)$ from s to the set U_c^L under neutral selection is same as the hitting time $H(cL, i)$ in the Markov chain on a line $M_{L,0}$, where s has exactly i-ones.

Each state has a self-loop with probability $(1-u)$, and we ignore the self-loop probabilities (i.e., set $u = 1$) because by Lemma S5 all lower bounds on the hitting time for the unloop variant are valid for the original Markov chain; and all upper bounds on the hitting time for the unloop variant need to be multiplied by $\frac{1}{u}$ to obtain the upper bounds on the hitting time for the original Markov chain. In other words, we will consider the following transition probabilities: (i) for $0 < i < L$ we have $\delta_0(i, i-1) = \frac{i}{L}$ and $\delta_0(i, i+1) = \frac{(L-i)}{L}$; (ii) $\delta_0(0, 1) = 1$; and (iii) $\delta_0(L, L-1) = 1$.

Theorem S1. *For all constants $c < \frac{1}{2}$, for all string spaces $B(L)$ with $L \geq \frac{4}{1-2 \cdot c}$, and for all $s \in B(L) \setminus U_c^L$, we have $T(U_c^L, s) \geq c_A^{\ell \cdot L - 1}$, where $A = \frac{3-2 \cdot c}{4} = \frac{1}{2} + \frac{1-2 \cdot c}{4} > \frac{1}{2}, c_A = \frac{A}{1-A} > 1$ and $\ell = \frac{1-2 \cdot c}{4} > 0$.*

Proof. We consider the Markov chain $M_{L,0}$ for $L \geq \frac{4}{1-2 \cdot c}$ and let us consider the midpoint i between cL and $\frac{1}{2} \cdot L$, i.e., $i = \frac{1+2 \cdot c}{4} \cdot L$. Such a midpoint exists since $L \geq \frac{4}{1-2 \cdot c}$. Then for all j such that $cL \leq j \leq i$ we have

$$\delta_0(j, j+1) = \frac{L-j}{L} \geq \frac{L-i}{L} = \frac{3 - 2 \cdot c}{4} = A > \frac{1}{2}.$$

The first inequality holds since $j \leq i$, while the second inequality is due to $c < \frac{1}{2}$. We now use Corollary S1 (item 1) for $M_{L,0}$ with $n_1 = x = cL$, $y = i$, and $k = (\frac{1+2 \cdot c}{4} - c) \cdot L = \ell \cdot L$ and vary n_2 from $x + 1$ to L to obtain that $H(n_1, n_2) \geq c_A^{\ell \cdot L - 1}$, and hence for all $s \in B(L) \setminus U_c^L$ we have $T(U_c^L, s) \geq c_A^{\ell \cdot L - 1}$. $\square$

Four-letter alphabet. As in typical cases in biology the alphabet size is four (e.g., DNA, RNA), we state the analog of Theorem S1 for a four-letter alphabet. Later in this document, Theorem S4 states the general case for arbitrary alphabet.

Consider the alphabet $\{0,1,2,3\}$. We can again consider a Markov chain on a line $M_{L,0}$, where its i-th position encodes all the strings in $B(L)$ which differ from t in exactly i positions. We consider a string s that corresponds to the i-th state of $M_{L,\beta}$, for $0 < i < L$. Then we have the following cases:

- There are exactly i neighbors of s in state $i-1$, since in each position among the i positions that s does not agree with t, there is exactly one mutation that will make s and t match in that position.
- There are exactly $3 \cdot (L-i)$ neighbors of s in state $i+1$, since in each position among the $L-i$ positions in which s agrees with t, there are 3 mutations that will make s not agree with t in that position.
- There are exactly $2 \cdot i$ neighbors of s in state i, since in each position j among the i positions that s does not agree with t, there are 2 mutations that will preserve this disagreement.

Based on the above analysis and Equation 1, the following holds for the transition probabilities of $M_{L,0}$:

$$\frac{\delta_0(i,i+1)}{\delta_0(i,i-1)} = \frac{3 \cdot (L-i)}{i}$$

while for $\delta_0(i,i)$ we have:

$$\delta_0(i,i) = \frac{2 \cdot i}{i + 3 \cdot (L-i) + 2 \cdot i} = \frac{1}{\frac{3}{2} \cdot \left(1 + \frac{L-i}{i}\right)}$$

which is maximized when $i = L$ to $\delta_0(L,L) = \frac{2}{3}$, constant.

Theorem S2. *For a four-letter alphabet size the following assertions hold :*

1. *if $c < \frac{3}{4}$, then there exists $L_0 \in \mathbb{N}$ such that for all string spaces $B(L)$ with $L \geq L_0$, for all $s \in B(L) \setminus U_c^L$ we have $T(U_c^L, s) \geq 2^{(3-4\cdot c)\frac{L}{16}\cdot\log\frac{6}{4+3\cdot c}}$; and*
2. *if $c \geq \frac{3}{4}$, then for all string spaces $B(L)$, for all $s \in B(L) \setminus U_c^L$ we have $T(U_c^L) = O(L^2)$.*

Proof. We prove each step separately.

1. We consider the Markov chain $M_{L,0}$ for $L \geq L_0 = \frac{8}{3-4\cdot c}$. Consider the midpoint i between cL and $\frac{3\cdot L}{4}$, i.e., $i = \frac{3+4\cdot c}{8} \cdot L$ (such a midpoint exists because $L \geq L_0$ and the choice of c). For all $cL < j \leq i$ we have:
$$\frac{\delta_\beta(j,j+1)}{\delta_\beta(j,j-1)} = \frac{3 \cdot (L-j)}{j} \geq \frac{3 \cdot (L-i)}{i} \geq 3 \cdot \frac{5-4\cdot c}{3+4\cdot c} \geq \frac{6}{4\cdot c+3} > 1$$
since $c < \frac{3}{4}$. We now use Lemma S3 for $M_{L,0}$ with $n_1 = x = cL$, $y = i$, and $k = L \cdot \frac{3-4\cdot c}{8} = \ell \cdot L$ and vary n_2 from $x+1$ to L to obtain that $H(n_1,n_2) \geq A^{\ell\cdot L-1}$, and hence for all $s \in B(L) \setminus U_c^L$ we have $T(U_c^L, s) \geq 2^{(\frac{3-4\cdot c}{8}\cdot L-1)\cdot\log\frac{6}{4+3\cdot c}} \geq 2^{(3-4\cdot c)\cdot\frac{L}{16}\cdot\log\frac{6}{4+3\cdot c}}$.

2. We consider the Markov chain $M_{L,0}$. For every $cL < j < L$ we have:
$$\frac{\delta_\beta(j,j+1)}{\delta_\beta(j,j-1)} = \frac{3 \cdot (L-j)}{j} \geq \frac{3 \cdot (L - c \cdot L)}{c \cdot L} = 1$$
Thus for all $cL < j < L$ we have $\delta_0(j,j-1) \geq \delta_\beta(j,j+1)$, and $\delta_0(j,j) \leq \frac{2}{3}$. Then, by Lemma S4 we have that $H(cL, n_2) = O(L^2)$ for all $n_2 > cL$, We conclude that $T(U_c^L, s) = O(L^2)$ for all $s \in B(s) \setminus U_c^L$.

□

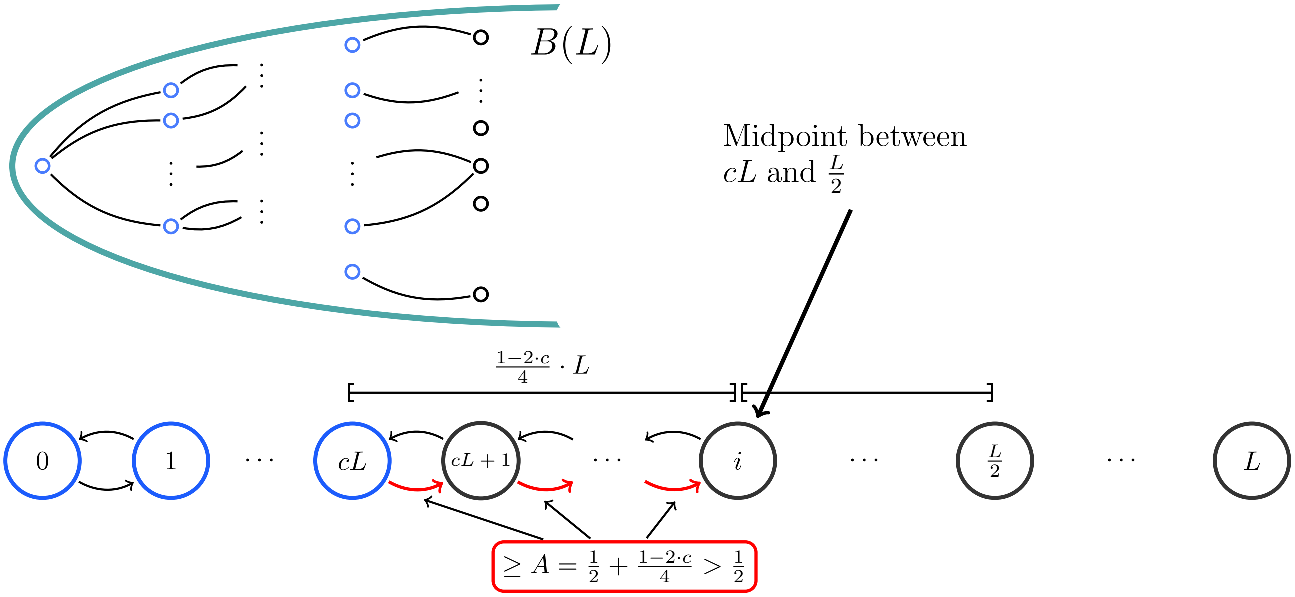

(a)

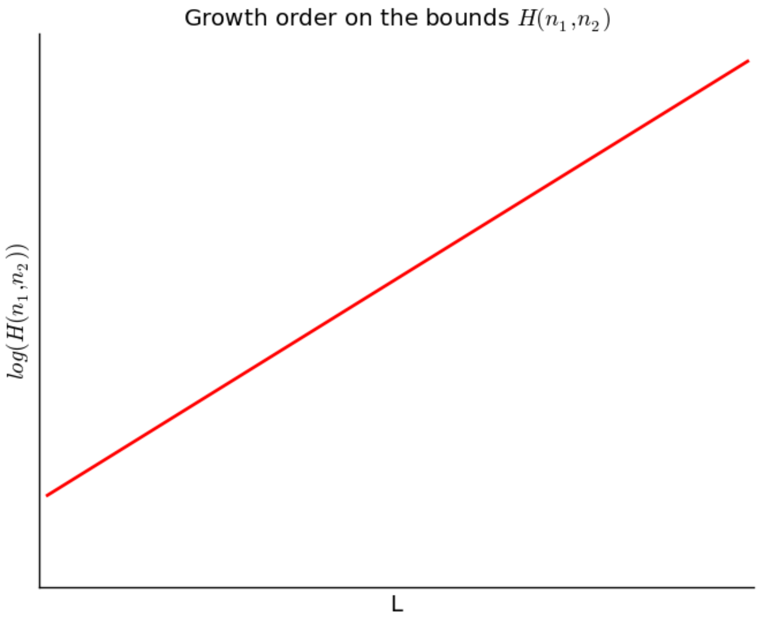

(b)

Supplementary Figure 3: Neutral selection with broad peaks. The figure shows that when the target set is U_c^L of strings that have at most $c \cdot n$ ones (blue in (a)), for $c < \frac{1}{2}$, for a region of length $\ell \cdot L - 1$, which is from $c \cdot n$ to the mid-point between cL and $\frac{L}{2}$, the transition probability to the right is at least a constant $A > \frac{1}{2}$, and this contributes to the exponential hitting time to the target set. Figure (b) shows the comparison of the exponential time for multiple targets and single target under neutral selection.

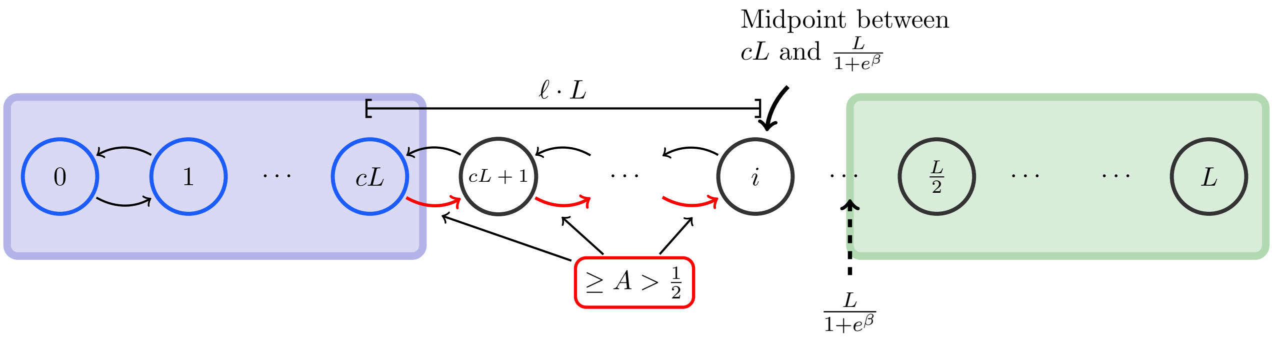

(a)

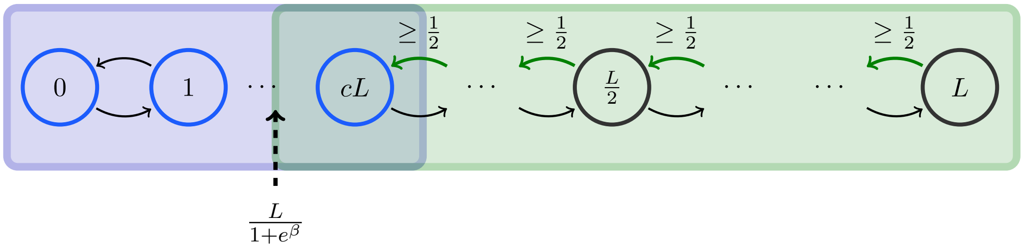

(b)

Supplementary Figure 4: Constant selection with broad peaks. The figure shows the illustration of the dichotomy theorem. The blue region represents the states that correspond to targets, while the green region depicts the states where the transition probability to the left is greater than $\frac{1}{2}$. Intuitively given a selection intensity β, the selection intensity allows to reach the region $\frac{1}{1+e^\beta} \cdot L$ in polynomial time. In figure (a), there exists a region between the blue and green, of length $\ell \cdot L$, where the probability of transitioning to the right is a constant, greater than $\frac{1}{2}$. In other words, when the blue and green region do not overlap, in the mid-region between the blue and green region the transition probability to the right is at least $A > \frac{1}{2}$, and hence the hitting time is exponential. When β and c are large enough so that the two regions overlap (figure (b)), then all transitions to the left till the target set is at least $\frac{1}{2}$, and hence the hitting time is polynomial.

5. Constant Fitness Difference Function

In this section we consider the case where the selection intensity $\beta > 0$ is positive, and the fitness function is linear. For a string s, let $h(s)$ denote the number of ones in s, i.e., the hamming distance from the string $\vec{0}$. We consider a linear fitness function f such that for two strings s and $s' \in \mathsf{Nh}(s)$ we have $df(s,s') = (f(s') - f(s)) = -(h(s') - h(s))$, the difference in the fitness is constant and depends negatively on the hamming distance. In other words, strings closer to $\vec{0}$ have greater fitness and the fitness change is linear with coefficient -1. We call the fitness function with constant difference as the *linear fitness function.* Again we consider a broad peak of targets U_c^L, for some constant $0 < c < \frac{1}{2}$. Since we consider all strings in U_c^L as the target set, it follows that for all strings $s \in B(L) \setminus U_c^L$ the difference in the hamming distance between s and $s' \in \mathsf{Nh}(s)$ from 0 and the target set U_c^L is the same. Similarly as in the neutral casel, due to symmetry of the linear fitness function f, we construct an equivalent Markov chain on a line, denoted $M_{L,\beta} = (S, \delta_\beta)$, as follows: state i of the Markov chain represents strings with exactly i-ones, and we have the following transition function: (i) $\delta_\beta(0,1) = 1$; (ii) $\delta_\beta(L, L-1) = 1$; and (iii) for $0 < i < L$ we have

$$\delta_\beta(i,i+1) = \frac{1}{1 + e^{\beta} \cdot \frac{i}{L-i}}; \qquad \delta_\beta(i,i-1) = \frac{1}{1 + e^{-\beta} \cdot \frac{L-i}{i}};$$

(also see the technical appendix for the derivation of the above probabilities).

Again the discovery time corresponds to the hitting time in the Markov chain $M_{L,\beta}$. Note that again we have ignored the self-loops of probability $(1-u)$, and by Lemma S5 all lower bounds for hitting time for the unloop variant are valid for the original Markov chain; and all upper bounds on the hitting time for the unloop variant need to be multiplied by $\frac{1}{u}$ to obtain upper bounds on the hitting time for the original Markov chain.

We will present a *dichotomy* result: the first result shows that if $c \cdot (1 + e^{\beta}) < 1$, for selection intensity $\beta > 0$, then the discovery time is exponential, while the second result shows that if $c \cdot (1 + e^{\beta}) \geq 1$, then the discovery time is polynomial. We first present the two lemmas.

Lemma S6. *For the linear fitness function f, for all selection intensities $\beta > 0$ and all constants $c \leq \frac{1}{2}$ such that $c \cdot v < 1$, where $v = 1 + e^{\beta}$, there exists $L_0 \in \mathbb{N}$ such that for all string spaces $B(L)$ with $L \geq L_0$, for all $s \in B(L) \setminus U_c^L$ we have $T(U_c^L, s, f, \beta) \geq c_A^{\ell \cdot L - 1}$ where $A = \frac{1}{2} + \frac{v \cdot (2 - c \cdot v)}{2 \cdot (v \cdot (c \cdot v + 2 - 2 \cdot c) - 2)} > \frac{1}{2}$, $c_A = \frac{A}{1-A} > 1$ and $\ell = \frac{1 - c \cdot v}{2 \cdot v} > 0$.*

Proof. We consider the Markov chain $M_{L,\beta}$ for $L \geq L_0 = \frac{2 \cdot v}{1 - c \cdot v}$. Consider the midpoint i between cL and $\frac{L}{v}$, i.e., $i = \frac{1 + c \cdot v}{2 \cdot v} \cdot L$ (such a midpoint exists because $L \geq L_0$ and the choice of c). For all $cL < j \leq i$ we have:

$$\begin{aligned}
\delta_\beta(j, j+1) &= \frac{1}{1 + e^{\beta} \cdot \frac{j}{L-j}} \geq \frac{1}{1 + e^{\beta} \cdot \frac{i}{L-i}} = \frac{1}{1 + (v-1) \cdot \frac{\frac{1+c \cdot v}{2 \cdot v} \cdot L}{L - \frac{1+c \cdot v}{2 \cdot v} \cdot L}} \\
&= \frac{2 \cdot v - 1 - c \cdot v}{c \cdot v^2 + 2 \cdot v \cdot (1-c) - 2} \\
&= \frac{1}{2} + \frac{v \cdot (2 - c \cdot v)}{2 \cdot (v \cdot (c \cdot v + 2 - 2 \cdot c) - 2)} = A > \frac{1}{2}.
\end{aligned}$$

The first inequality holds as $\frac{j}{L-j} \leq \frac{i}{L-i}$ since $j \leq i$; the second equality is obtained since $(v-1) = e^{\beta}$ and substituting i with its value $\frac{1+c \cdot v}{2 \cdot v} \cdot L$; and the result of the equalities are simple calculation; and the description of the final inequality is as follows: (i) since $c \cdot v < 1$, we have $2 - c \cdot v > 0$, (ii) the fact that $c \leq \frac{1}{2}$ and $c \cdot v \geq 0$ implies that $c \cdot v + 2 - 2 \cdot c \geq 1$ and since we have $v > 2$, it follows that $v \cdot (c \cdot v + 2 - 2 \cdot c) > 2$; establishing that the term along with $\frac{1}{2}$ in A is strictly positive. We now use Corollary S1 (item 1) for $M_{L,\beta}$ with $n_1 = x = cL$, $y = i$, and $k = \frac{1 - c \cdot v}{2 \cdot v} \cdot L = \ell \cdot L$ and vary n_2 from $x+1$ to L to obtain that $H(n_1, n_2) \geq c_A^{\ell \cdot L - 1}$, and hence for all $s \in B(L) \setminus U_c^L$ we have $T(U_c^L, s, f, \beta) \geq c_A^{\ell \cdot L - 1}$. □

Lemma S7. *For all string spaces $B(L)$, for all $c < \frac{1}{2}$ and the linear fitness function, for all selection intensities $\beta > 0$ with $c \cdot (1 + e^{\beta}) \geq 1$, for all $s \in B(L) \setminus U_c^L$ we have $T(U_c^L, s, f, \beta) = O(L^2)$.*

Proof. We consider the Markov chain $M_{L,\beta}$, where β is such that we have $c \geq \frac{1}{1+e^{\beta}}$. For every $cL < j < L$ we have:

$$\delta_\beta(j, j-1) = \frac{1}{1 + e^{-\beta} \cdot \frac{L-j}{j}} \geq \frac{1}{1 + e^{-\beta} \cdot \frac{L-cL}{cL}} = \frac{1}{1 + e^{-\beta} \cdot \frac{1-c}{c}} \geq \frac{1}{2}.$$

The first inequality holds because $\frac{L-j}{j} \leq \frac{L-cL}{cL}$ since $cL < j$; the second inequality holds since $c \cdot (1+e^{\beta}) \geq 1$ which implies that $1 \geq \frac{1}{e^{\beta}} \cdot (\frac{1}{c} - 1)$, and hence $1 + e^{-\beta} \cdot (\frac{1}{c} - 1) \leq 2$. Thus for all $cL < j < L$ we have $\delta_\beta(j, j-1) \geq \frac{1}{2}$, and by Corollary S1 (item 2) we have that $H(cL, n_2) = O(L^2)$ for all $n_2 > cL$. Thus we conclude that $T(U_c^L, s, f, \beta) = O(L^2)$ for all $s \in B(s) \setminus U_c^L$. The desired result follows. $\square$

Theorem S3. *For the linear fitness function f, selection intensity $\beta > 0$, and constant $c \leq \frac{1}{2}$, the following assertions hold:*

1. *If $c \cdot (1 + e^{\beta}) < 1$, then there exists $L_0 \in \mathbb{N}$ such that for all string spaces $B(L)$ with $L \geq L_0$, for all $s \in B(L) \setminus U_c^L$ we have $T(U_c^L, s, f, \beta) \geq c_A^{\ell \cdot L - 1}$ where $A = \frac{1}{2} + \frac{v \cdot (2 - c \cdot v)}{2 \cdot (v \cdot (c \cdot v + 2 - 2 \cdot c) - 2)} > \frac{1}{2}$, $c_A = \frac{A}{1-A} > 1$ and $\ell = \frac{1 - c \cdot v}{2 \cdot v} > 0$.*
2. *If $c \cdot (1+e^{\beta}) \geq 1$, then for all string spaces $B(L)$, for all $s \in B(L) \setminus U_c^L$ we have $T(U_c^L, s, f, \beta) = O(L^2)$.*

6. Moran Process Model

In the previous section we considered the constant selection intensity with Fermi process. We now discuss how from the results of the previous section we can obtain similar results if we consider the Moran process for evolution.

Basic Moran process description. A population of N individuals mutates with probability u in each round, at $N \cdot u$ rate. Consider that the population is currently in state i (which represents all bit strings with exactly i ones): the probability that the next state is $i - 1$ is the rate of an $i - 1$ mutant to be introduced, times the fixation probability of the mutant in the population. Formally, the transition probability matrix δ_M (M for Moran process) for the Markov chain on a line under the Moran process is as follows:

(1) $\delta_M(i, i-1) = N \cdot u \cdot \frac{i}{L} \cdot \rho_{i,i-1}$;   (2) $\delta_M(i, i+1) = N \cdot u \cdot \frac{L-i}{L} \cdot \rho_{i,i+1}$;   (3) $\delta_M(i, i) = 1 - \delta_M(i, i-1) - \delta_M(i, i+1)$.

We assume that $N \cdot u < 1$ and $\rho_{i,j}$ is the fixation probability of a j mutant in a population of $N - 1$ individuals of type i. In particular,

$$\rho_{i,j} = \frac{1 - \frac{f_i}{f_j}}{1 - \left(\frac{f_i}{f_j}\right)^N}$$

and $\rho_{i,j} \in (0, 1)$ for positive fitness f_i and f_j, where f_i (resp. f_j) denotes the fitness of strings with exactly i (resp. j) ones. We first show a bound for the self-loop probabilities $\delta_M(i, i)$: since strings closer to the target have a greater fitness value we have $f_{i-1} \geq f_i$; and hence the probability of fixation of an $(i - 1)$-mutant in a population of type i is at least $\frac{1}{N}$. Thus we have

$$\delta_M(i, i-1) = N \cdot u \cdot \frac{i}{L} \cdot \rho_{i,i-1} \geq N \cdot u \cdot \frac{i}{L} \cdot \frac{1}{N} \geq \frac{u}{L}$$

Then, $1 - \delta_M(i, i) \geq \delta_M(i, i-1) \geq \frac{u}{L}$, and $\frac{1}{1-\delta_M(i,i)} \leq \frac{L}{u}$. Hence we will consider the unloop variant of the Markov chain and by Lemma S5 all lower bounds on discovery time for the unloop variant hold for the original Markov chain; and the upper bounds for the unloop variant need to by multiplied by $\frac{L}{u}$ to obtain

the upper bounds for the original Markov chain. Hence if we consider the unloop variant of the Markov chain on a line obtained from the Moran process we have:

$$\overline{\delta}_M(i,i-1) = \frac{\delta_M(i,i-1)}{\delta_M(i,i-1)+\delta_M(i,i+1)} = \frac{i}{i+(L-i)\cdot\frac{\rho_{i,i+1}}{\rho_{i,i-i}}} = \frac{1}{1+\frac{L-i}{i}\cdot\frac{\rho_{i,i+1}}{\rho_{i,i-i}}}$$

and $\overline{\delta}_M(i,i+1) = 1-\overline{\delta}_M(i,i-1)$. We now consider the case of multiplicative fitness function.

Multiplicative fitness rates. We consider the case where we have multiplicative fitness function where $\frac{f_{i-1}}{f_i} = r_i \geq 1$, as the fitness function increases as we move closer to the target. Then

$$\frac{\rho_{i,i+1}}{\rho_{i,i-1}} = \frac{\frac{1-r_{i+1}}{1-r_{i+1}^N}}{\frac{1-r_i^{-1}}{1-r_i^{-N}}} = r_i^{-(N-1)}\cdot\frac{r_i^N-1}{r_{i+1}^N-1}\cdot\frac{r_{i+1}-1}{r_i-1}$$

and

$$\overline{\delta}_M(i,i-1) = \frac{1}{1+\frac{L-i}{i}\cdot\frac{\rho_{i,i+1}}{\rho_{i,i-1}}} = \frac{1}{1+\frac{L-i}{i}\cdot r_i^{-(N-1)}\cdot\frac{r_i^N-1}{r_{i+1}^N-1}\cdot\frac{r_{i+1}-1}{r_i-1}}.$$

For constant factor $r_i = r$ for all i, we obtain

$$\overline{\delta}_M(i,i-1) = \frac{1}{1+\frac{L-i}{i}\cdot r^{-(N-1)}}.$$

Let us denote by $\overline{\delta}_{M,r}(i,i-1) = \frac{1}{1+\frac{L-i}{i}\cdot r^{-(N-1)}}$ the transition probabilities of the unloop variant of the Markov chain on a line for the Moran process with multiplicative constant r. Then we have the following cases:

1. *(Neutral case).* In the neutral case we have $r=1$, and then the Markov chain with transition probabilities $\overline{\delta}_{M,1}$ is the same as the transition probabilities δ_0 of the Markov chain $M_{L,0}$ in Section 4 for neutral selection.

2. *(Constant r multiplicative fitness).* The transition probabilities $\overline{\delta}_{M,r}(i,i-1)$ has the same form as the transition probabilities of the Markov chain $M_{L,\beta}$ under positive selection intensity and linear fitness function, in Section 5. In particular, for $e^\beta = r^{N-1}$, we have δ_β of $M_{L,\beta}$ is the same as $\overline{\delta}_{M,r}$, and thus from the results of Section 5 we obtain similar results for the Moran process.

Summary of results for Moran process with multiplicative fitness landscape. From the results of Section 4 and Section 5, and the equivalence of the transition probabilities of the Markov chains in Section 4 and Section 5 with those in the Moran process, we obtain the following results for Moran process of evolution under constant multiplicative fitness landscape r:

1. *(Single target).* For a single target, for all constants r and population size N, the discovery time from any non-target string to the target is exponential in the length of the bit strings.

2. *(Broad peaks).* For broad peaks with constant c fraction of clustered targets with $c \leq \frac{1}{2}$, if $c\cdot(1+r^{N-1}) < 1$, then the discovery time from any non-target string to the target set is at least exponential in the length L of the bit strings; and if $c\cdot(1+r^{N-1}) \geq 1$, then the discovery time from any non-target string to the target set is at most $O(\frac{L^3}{u})$ (i.e., polynomial).

The polynomial discovery time for a broad peak surrounded by a fitness slope, requires the slope to extend to a Hamming distance greater than $3L/4$. What happens then, if the slope only extends to a certain maximum distance less than $3L/4$? Suppose the fitness gain only arises, if the sequence differs from the specific sequence in not more than a fraction s of positions. Formally, we can consider any fitness function, f, that assigns zero fitness to sequences that are at a Hamming distance of at least sL from the specific

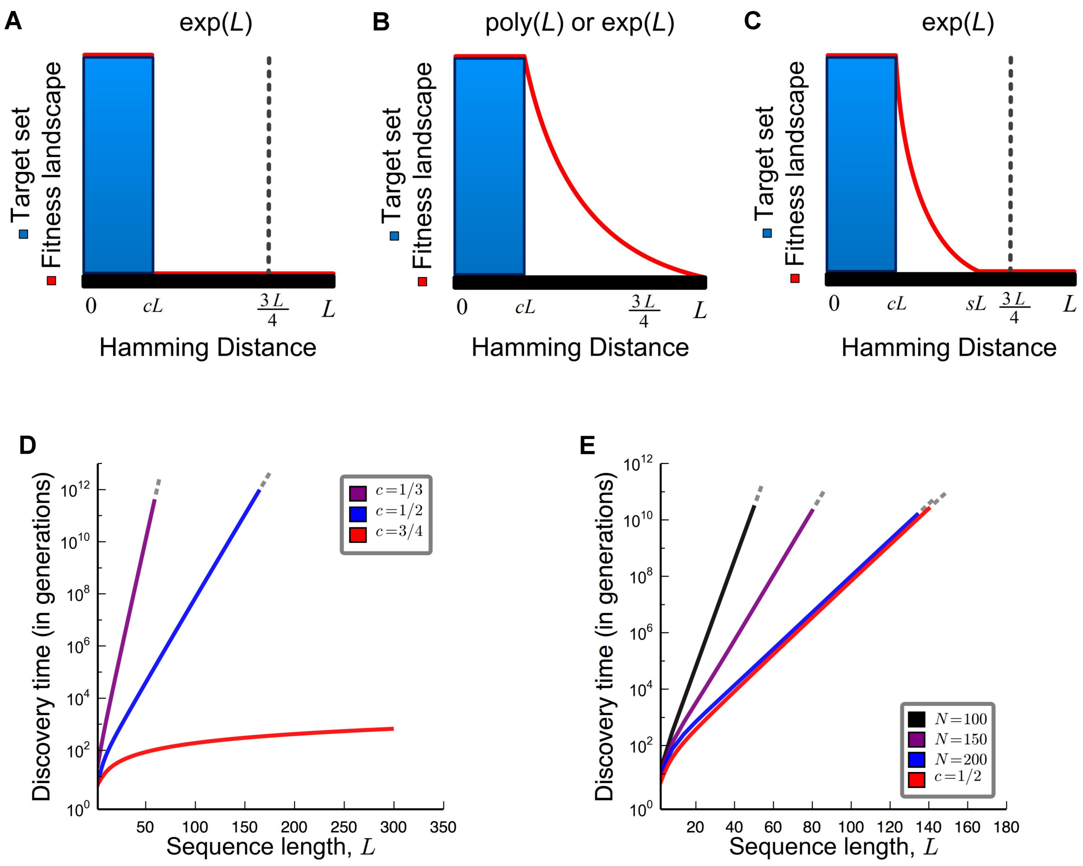

Supplementary Figure 5: Broad peak with different fitness landscapes. For the broad peak there is a specific sequence, and all sequences that are within Hamming distance cL are part of the target set. (A) If the fitness landscape is flat outside the broad peak and if $c < 3/4$, then the discovery time is exponential in sequence length, L. (B) If the broad peak is surrounded by a multiplicative fitness landscape whose slope extends over the whole sequence space, then the discovery time is either polynomial or exponential in L depending on whether $c(1 + r^{N-1}/3) \geq 1$ or not. (C) If the fitness slope extends to a Hamming distance less than $3L/4$, then the discovery time is exponential in L. (D) Numerical calculations for broad peaks surrounded by flat fitness landscapes. We observe exponential discovery time for $c = 1/3$ and $c = 1/2$. (E) Numerical calculations for broad peaks surrounded by multiplicative fitness landscapes. The broad peak extends to $c = 1/6$ and the slope of the fitness landscape to $s = 1/2$. The discovery time is exponential, because $s < 3/4$. The fitness gain is $r = 1.01$ and the population size is as indicated. As the population size, N, increases the discovery time converges to that of a broad peak with $c = 1/2$ embedded in a flat fitness landscape.

sequence. Now our previous result for neutral drift with broad peak applies. Since we must rely on neutral drift until the fitness gain arises, the discovery time in this fitness landscape is at least as long as the discovery time for neutral drift with a broad peak of size $c = s$. If $s < 3/4$, then the expected discovery time starting from any sequence outside the fitness gain region is exponential in L. Tables 1 and 2 illustrate how setting $c = s$ under-approximates the expected discovery time.

$r = 1.01$		$N = 10^2$	$N = 5 \cdot 10^2$	$N = 10^3$	$N = 10^4$	$N = \infty$
$s = \frac{1}{3}$	$c = \frac{1}{12}$	$1.872592 \cdot 10^{337}$	$6.149382 \cdot 10^{170}$	$5.893335 \cdot 10^{170}$	$5.891566 \cdot 10^{170}$	$5.891566 \cdot 10^{170}$
	$c = \frac{1}{6}$	$5.962263 \cdot 10^{260}$	$6.149382 \cdot 10^{170}$	$5.893335 \cdot 10^{170}$	$5.891566 \cdot 10^{170}$	$5.891566 \cdot 10^{170}$
$s = \frac{1}{2}$	$c = \frac{1}{12}$	$3.285017 \cdot 10^{264}$	$1.307607 \cdot 10^{65}$	$1.285938 \cdot 10^{65}$	$1.285790 \cdot 10^{65}$	$1.285790 \cdot 10^{65}$
	$c = \frac{1}{6}$	$1.396805 \cdot 10^{188}$	$1.307607 \cdot 10^{65}$	$1.285938 \cdot 10^{65}$	$1.285790 \cdot 10^{65}$	$1.285790 \cdot 10^{65}$

Supplementary Table 1: Numerical data for the discovery time of broad peaks embedded in multiplicative fitness landscapes. The width of the broad peak is either $c = 1/12$ or $c = 1/6$ and $L = 1000$. The fitness slope extends to $s = 1/3$ and $s = 1/2$. The data are extrapolated from numbers obtained for small values of L. For population sizes $N = 1000$ and greater, there is no difference in the discovery time of $c = 1/6$ and $c = 1/12$. For $N \to \infty$ the discovery time for a particular s converges to the discovery time for a broad peak with $c = s$ embedded in a flat fitness landscape.

Figure 5 gives a pictorial illustration of all the above scenarios.

7. General Alphabet

In previous sections we presented our results for L-bit strings. In this section, we consider the case of general alphabet, where every sequence consists of letters from a finite alphabet Σ. Thus, $B(L)$ is the space of all L-tuple strings in Σ^L. We fix a letter $\sigma \in \Sigma$, and consider a target set U_c^L, consisting of all the L-tuple strings, such that every $s \in U_c^L$ differs from the target string $t = \sigma^L$ (of all σ's) in at most cL positions (i.e., Hamming distance at most $c \cdot L$ from the target string t). We will prove a dichotomy result that generalizes Theorem S3.

We can again consider a Markov chain on a line $M_{L,\beta}$, where its i-th position encodes all the strings in $B(L)$ which differ from t in exactly i positions. We consider a string s that corresponds to the i-th state of $M_{L,\beta}$, for $0 < i < L$. Then we have the following cases:

- There are exactly i neighbors of s in state $i - 1$, since in each position among the i positions that s does not agree with t, there is exactly one mutation that will make s and t match in that position.
- There are exactly $(L - i) \cdot (|\Sigma| - 1)$ neighbors of s in state $i + 1$, since in each position among the $L - i$ positions in which s agrees with t, there are $|\Sigma| - 1$ mutations that will make s not agree with t in that position.
- There are exactly $i \cdot (|\Sigma| - 2)$ neighbors of s in state i, since in each position j among the i positions that s does not agree with t, there are $|\Sigma| - 2$ mutations that will preserve this disagreement.

Let us denote $|\Sigma| = 1 + \kappa$, where $\kappa \geq 1$. Based on the above analysis and Equation 1, the following holds for the transition probabilities of $M_{L,\beta}$:

$$\frac{\delta_\beta(i,i+1)}{\delta_\beta(i,i-1)} = \frac{(L-i)\cdot\kappa\cdot\frac{1}{1+e^{\beta}}}{\frac{i}{1+e^{-\beta}}} = \frac{L-i}{i}\cdot\kappa\cdot e^{-\beta}$$

while for $\delta_\beta(i,i)$ we have:

- *($\kappa = 1$):* Then $\delta_\beta(i,i) = 0$, since every mutation changes the distance from t.
- *($\kappa > 1$):* Then by Equation 1, for $0 < i \le L$:

$$\delta_\beta(i,i) = \frac{1}{1 + \frac{2}{(\kappa-1)\cdot(1+e^{-\beta})} + \frac{2\cdot(L-i)\cdot(\kappa)}{i\cdot(\kappa-1)\cdot(1+e^{\beta})}}$$

which is maximized when $i = L$ to $\delta_\beta(L,L) = \frac{1}{1+\frac{2}{(\kappa-1)\cdot(1+e^{-\beta})}}$, constant for a fixed alphabet Σ.

Lemma S8. *For the linear fitness function f, for all selection intensities $\beta \ge 0$ and all constants $c \le \frac{\kappa}{\kappa+1}$ such that $c\cdot v < 1$ for $v = 1+\frac{e^{\beta}}{\kappa}$, there exists $L_0 \in \mathbb{N}$ such that for all string spaces $B(L)$ with $L \ge L_0$, for all $s \in B(L)\setminus U_c^L$ we have $T(U_c^L, s, f, \beta) \ge A^{\ell\cdot L-1}$, where $A = \frac{v\cdot(2-c)-1}{1+c\cdot v}\cdot\kappa\cdot e^{-\beta} > 1$ and $\ell = \frac{1-c\cdot v}{2\cdot v}$.*

Proof. We consider the Markov chain $M_{L,\beta}$ for $L \ge L_0 = \lceil \frac{2\cdot v}{1-c\cdot v} \rceil$. Consider the midpoint i between cL and $\frac{L}{v}$, i.e., $i = L\cdot\frac{1+c\cdot v}{2\cdot v}$ (such a midpoint exists because $L \ge L_0$ and the choice of c, as $i > cL$). For all $cL < j \le i$ we have:

$$\frac{\delta_\beta(j,j+1)}{\delta_\beta(j,j-1)} \;=\; \frac{L-j}{j}\cdot\kappa\cdot e^{-\beta} \ge \frac{L-i}{i}\cdot\kappa\cdot e^{-\beta} = \frac{L-L\cdot\frac{1+c\cdot v}{2\cdot v}}{L\cdot\frac{1+c\cdot v}{2\cdot v}}\cdot\kappa\cdot e^{-\beta} = \frac{2\cdot v-1-c\cdot v}{1+c\cdot v}\cdot\kappa\cdot e^{-\beta} = A > 1$$

The first inequality holds because $j \le i$ and thus $\frac{L-j}{j} \ge \frac{L-i}{i}$. The equalities follow as simple rewriting, while $A > \frac{2\cdot v-2}{2}\cdot\kappa\cdot e^{-\beta} = (v-1)\cdot\kappa\cdot e^{-\beta} = 1$, since $c\cdot v < 1$. We now use Lemma S3 for $M_{L,\beta}$ with $n_1 = x = cL$, $y = i$, and $k = L\cdot\frac{1-c\cdot v}{2\cdot v} = \ell\cdot L$ and vary n_2 from $x+1$ to L to obtain that $H(n_1,n_2) \ge A^{\ell\cdot L-1}$, and hence for all $s \in B(L)\setminus U_c^L$ we have $T(U_c^L, s, f, \beta) \ge A^{\ell\cdot L-1}$. □

Lemma S9. *For all string spaces $B(L)$, for all $c \le \frac{\kappa}{\kappa+1}$ and the linear fitness function f, for all selection intensities $\beta \ge 0$ with $c\cdot v \ge 1$ for $v = 1+\frac{e^{\beta}}{\kappa}$, for all $s \in B(L)\setminus U_c^L$ we have $T(U_c^L, s, f, \beta) = O(\frac{L^2}{M})$, where $M = \min_{0<i\le L} 1-\delta_\beta(i,i) = 1-\frac{1}{1+\frac{2}{(\kappa-1)\cdot(1+e^{-\beta})}}$.*

Proof. We consider the Markov chain $M_{L,\beta}$, where β is such that we have $c\cdot v \ge 1$. For every $cL < j < L$ we have:

$$\frac{\delta_\beta(j,j-1)}{\delta_\beta(j,j+1)} = \frac{j}{L-j}\cdot\frac{e^{\beta}}{\kappa} \ge \frac{cL}{L\cdot\kappa\cdot(1-c)}\cdot e^{\beta} = \frac{c\cdot e^{\beta}}{\kappa-c\cdot\kappa} \ge 1$$

The first inequality holds because $cL < j$; the second inequality holds because $c\cdot(1+\frac{e^{\beta}}{\kappa}) \ge 1$ and thus $\frac{c\cdot e^{\beta}}{\kappa-c\cdot\kappa} \ge 1$. Thus for all $cL < j < L$ we have $\delta_\beta(j,j-1) \ge \delta_\beta(j,j+1)$, while $M = \min_{0<i\le L} 1-\delta_\beta(i,i) = 1-\frac{1}{1+\frac{2}{(\kappa-1)\cdot(1+e^{-\beta})}}$. Then, by Lemma S4 we have that $H(cL, n_2) = O(\frac{L^2}{M})$ for all $n_2 > cL$. We conclude that $T(U_c^L, s, f, \beta) = O(\frac{L^2}{M})$ for all $s \in B(s)\setminus U_c^L$. The desired result follows. □

Lemmas S8 and S9 yield the following dichotomy (recall that $|\Sigma| = 1+\kappa$):

Theorem S4. *For alphabet size $|\Sigma|$, for the linear fitness function f, selection intensity $\beta \ge 0$, and constant $c \le \frac{\kappa}{\kappa+1}$, where $|\Sigma| = 1+\kappa$; the following assertions hold :*

1. *if $c\cdot(1+\frac{e^{\beta}}{\kappa}) < 1$, then there exists $L_0 \in \mathbb{N}$ such that for all string spaces $B(L)$ with $L \ge L_0$, for all $s \in B(L)\setminus U_c^L$ we have $T(U_c^L, s, f, \beta) \ge A^{\ell\cdot L-1}$ where $A = \frac{v\cdot(2-c)-1}{1+c\cdot v}\cdot\kappa\cdot e^{-\beta} > 1$ and $\ell = \frac{1-c\cdot v}{2\cdot v}$, with $v = \frac{e^{\beta}+\kappa}{\kappa}$; and*

2. *if* $c\cdot(1+\frac{e^{\beta}}{\kappa})\geq 1$*, then for all string spaces* $B(L)$*, for all* $s\in B(L)\setminus U_c^L$ *we have* $T(U_c^L,s,f,\beta)=O(\frac{L^2}{M})$*, where* $M=1-\frac{1}{1+\frac{2}{(\kappa-1)\cdot(1+e^{-\beta})}}$*.*

Note that Theorem S4 with the special case of $|\Sigma|=2$ and $\kappa=1$ gives us Theorem S3.

Corollary S2. *For alphabet size* $|\Sigma|=1+\kappa$*, consider the Moran process with multiplicative fitness landscape with constant* r*, population size* N*, and mutation rate* u*. Let* $c\leq\frac{\kappa}{\kappa+1}$*. The following assertions hold :*

1. *if* $c\cdot(1+\frac{r^{N-1}}{\kappa})<1$*, then there exists* $L_0\in\mathbb{N}$ *such that for all string spaces* $B(L)$ *with* $L\geq L_0$*, for all* $s\in B(L)\setminus U_c^L$ *the discovery time from* s *to some string in* U_c^L *is at least* $A^{\ell\cdot L-1}$ *where* $A=\frac{v\cdot(2-c)-1}{1+c\cdot v}\cdot\kappa\cdot r^{1-N}>1$ *and* $\ell=\frac{1-c\cdot v}{2\cdot v}$*, with* $v=1+\frac{r^{N-1}}{\kappa}$*; and*

2. *if* $c\cdot(1+\frac{r^{N-1}}{\kappa})\geq 1$*, then for all string spaces* $B(L)$*, for all* $s\in B(L)\setminus U_c^L$ *the discovery time from* s *to some string in* U_c^L *is at most* $O(\frac{L^3}{M\cdot u})$*, where* $M=1-\frac{1}{1+\frac{2}{(\kappa-1)\cdot(1+r^{-(N-1)})}}$ *is constant.*

Explicit bounds for four letter alphabet. We now present the explicit calculation for L_0 and ℓ of Corollary S2 for four letter alphabet. For the four letter alphabet we have $\kappa=3$, and for the exponential lower bound we have $cv<1$. In this case we have

$$v=\frac{3+r^{N-1}}{3}\qquad\text{and}\qquad\ell=\frac{1-3c-cr^{N-1}}{6+2r^{N-1}}=\frac{3(1-c)-cr^{N-1}}{6+2r^{N-1}}.$$

Since $cv<1$ we have

$$A=3r^{1-N}\frac{2v-cv-1}{1+cv}\geq\frac{2(v-1)}{1+cv}=3r^{1-N}\frac{2\frac{r^{N-1}}{3}}{\frac{3+3c+cr^{N-1}}{3}}=\frac{6}{3(1+c)+cr^{N-1}}$$

By changing the exponential lower bound to base 2, we have that the discovery time is at least $2^{(\ell L-1)\log_2 A}$. Thus we have the following two cases:

- *Selection:* With selection (i.e., $r>1$) the exponential lower bound on the discovery time when $cv<1$ is at least:

$$2^{\left(\frac{3(1-c)-cr^{N-1}}{6+2r^{N-1}}L-1\right)\log_2\frac{6}{3(c+1)+cr^{N-1}}};$$

  for all $L\geq L_0=\frac{6+2r^{N-1}}{3(1-c)-cr^{N-1}}$.

- *Neutral case:* Specializing the above result for the neutral case (i.e., $r=1$) we obtain the exponential lower bound on the discovery time when $cv<1$ is at least:

$$2^{\left(\frac{3-4c}{8}L-1\right)\log_2\frac{6}{4c+3}};$$

  for all $L\geq L_0=\frac{8}{3-4c}$. We ignore the factor 1 as compared to L and have that $2^{\left(\frac{3-4c}{8}L-1\right)\log_2\frac{6}{4c+3}}\geq\exp\left(\left(\frac{3-4c}{16}L\right)\log_2\frac{6}{4c+3}\right)$.

Discussion about implications of results. We now discuss the implications of Corollary S2.

1. First the corollary implies that for a single target (which intuitively corresponds to $c=0$) even with multiplicative fitness landscape (which is an exponentially increasing fitness landscape) the discovery time is exponential.
2. The discovery time is polynomial if $c\cdot(1+\frac{r^{N-1}}{\kappa})\geq 1$, however this requires that the slope of the fitness gain extends over the whole sequence space (at least till Hamming distance $(\kappa/(\kappa+1))\cdot L$).

3. Consider the case where the fitness gain arises only when the sequence differs from the target in not more than a fraction of s positions, i.e., the slope of the fitness function only extends upto a Hamming distance of $s \cdot L$. Now our result for neutral drift with broad peak applies. Since we must rely on neutral drift until the fitness gain arises, the discovery time of this process is at least as long as the discovery time for neutral drift with a broad peak of size $c = s$. If $r = 1$ (neutral drift), then we have that the discovery time is polynomial if $c(1 + \frac{1}{\kappa}) \geq 1$, and otherwise it is exponential. Hence if the fitness gain arises from Hamming distance $s \cdot L$ and $s < \kappa/(\kappa + 1)$, then the expected discovery time starting from any sequence outside the fitness gain region is exponential in L. Moreover, there are two further implications of this exponential lower bound. First, note that if $r = 1$, then r^{N-1} is 1 independent of N, and thus the exponential lower bound is independent of N. Second, note that if the fitness gain arises from Hamming distance $s \cdot L$, and it is neutral till the fitness gain region is reached, then the exponential lower bound for $s < \kappa/(\kappa + 1)$, is also independent of the shape of the fitness landscape after the fitness gain arises. Formally, if we consider any fitness function f that assigns zero fitness to strings that are at Hamming distance at least $s \cdot L$ from the ideal sequence, and any nonnegative fitness value to other strings, then the process is neutral till the fitness gain arises, and the exponential lower bound holds for the fitness landscape, and is independent of the population size. For a four letter alphabet (as in the case of RNA and DNA) the critical threshold is thus $s = 3/4$.

Remark S1. Note that we have shown that all results for bit strings easily extend to any finite alphabet by appropriately changing the constant. For simplicity, in the following sections we present our results for strings over 4-letter alphabet, and they also extend easily to any finite alphabet by appropriately changing the constants.

Remark S2. We have established several lower bounds on the expected discovery time. All the lower bounds are obtained from hitting times on Markov chains, and in Markov chains the hitting times are closely concentrated around the expectation. In other words, whenever we establish that the expected discovery time is exponential, it follows that the discovery time is exponential with high probability.

8. Multiple Independent Searches

In this section we consider multiple independent searches. For simplicity we will consider strings over 4-letter alphabet, and as shown in Section 7 the results easily extend to strings over alphabets of any size.

8.1. Polynomially many independent searches. We will show that if there are polynomially many multiple searches starting from a Hamming distance of at least $\frac{3L}{4}$, then the probability to reach the target in polynomially many steps is negligibly small (smaller than an inverse of any polynomial function). We will present our results for Markov chain on a line, and it implies the results for the evolutionary processes. In all the following lemmas we consider the Markov chain on a line for a four letter alphabet. Before the formal proof we present informal arguments and intuition for the proof.

The basic intuition and steps of the proof. The basic intuition and steps of our proof are as follows:

1. First we show that in the Markov chain on a line, from any point $n_2 \geq \frac{3L}{4}$ the probability to not reach $\frac{3L}{4}$ in polynomially many steps is very (exponentially) small. The key reason is that we have shown that the expected hitting time from n_2 to $\frac{3L}{4}$ is at most L^2; and hence the probability to reach $\frac{3L}{4}$ from n_2 within L^5 steps is very high. Thus the probability to not reach within L^5 steps is very small (see Lemma S10).

2. Second, we show that the contribution to the expected hitting time for the steps beyond $L^2 \cdot 2^{L \log L}$ is at most a constant. The informal reasoning is that beyond the expected hitting time, the probability to not reach in steps beyond the expected hitting time drops exponentially. Hence we obtain a geometric series whose sum is bounded by a constant (see Lemma S11).

3. Then we show that if the expected hitting time is exponential, then for all polynomials $p_1(\cdot)$ and $p_2(\cdot)$, the probability to reach within $p_1(L)$ steps is smaller than $\frac{1}{p_2(L)}$. The key argument is to combine the previous two results to show that if the probability to reach within $p_1(L)$ steps is more than $\frac{1}{p_2(L)}$, then the expected hitting time would be a polynomial, contradicting that the expected hitting time is exponential.

The formal arguments of the above results yield Theorem S5. We present the formal proof below.

Lemma S10. *From any point $n_2 \geq \frac{3L}{4}$ the probability that $\frac{3L}{4}$ is not reached within L^5 steps is exponentially small in L (i.e., at most e^{-L}).*

Proof. We have already established that the expected hitting time from n_2 to $\frac{3L}{4}$ is at most L^2. Hence the probability to reach $\frac{3L}{4}$ within L^3 steps must be at least $\frac{1}{L}$ (otherwise the expectation would have been greater than L^2). Since from all states $n_2 \geq \frac{3L}{4}$ the probability to reach $\frac{3L}{4}$ is at least $\frac{1}{L}$ within L^3 steps, the probability that $\frac{3L}{4}$ is not reached within $k \cdot L^3$ steps is at most $\left(1 - \frac{1}{L}\right)^k$. Hence the probability that $\frac{3L}{4}$ is not reached within L^5 steps is at most

$$\left(1 - \frac{1}{L}\right)^{L \cdot L} \leq e^{-L}.$$

The desired result follows. □

Lemma S11. *The contribution of the expectation to reach after $L^2 \cdot 2^{L \cdot \log L}$ steps to the expected hitting time is at most a constant (i.e., $O(1)$).*

Proof. From any starting point, the probability to reach the target within L steps is at least $\frac{1}{L^L}$. Hence the probability not reaching the target within $k \cdot L^L$ steps is e^{-k}. Hence the probability to reach after $\ell \cdot L^2 \cdot 2^{L \cdot \log L}$ steps at most $e^{-\ell \cdot L^2}$. Thus expectation contribution from $L^2 \cdot 2^{L \cdot \log L}$ steps is at most

$$\sum_{\ell=1}^{\infty} \frac{(\ell+1) \cdot L^2 \cdot 2^{L \cdot \log L}}{e^{\ell \cdot L^2}} \leq \frac{L^2 \cdot 2^{L \cdot \log L}}{e^{L^2}} \cdot \sum_{\ell=1}^{\infty} \frac{(\ell+1)}{e^{\ell}} \leq \frac{2^{2 \cdot \log L + L \cdot \log L}}{2^{L^2}} \cdot \sum_{\ell=1}^{\infty} \left(\frac{\ell}{e^{\ell}} + \frac{1}{e^{\ell}}\right) \leq \frac{e}{(e-1)^2} + \frac{1}{(e-1)} = O(1).$$

The desired result follows. □

Lemma S12. *In all cases, where the lower bound on the expected hitting time is exponential, for all polynomials $p_1(\cdot)$ and $p_2(\cdot)$, the probability to reach the target set from any state n_2 such that $n_2 \geq \frac{3L}{4}$ within the first $p_1(L)$ steps is at most $\frac{1}{p_2(L)}$.*

Proof. We first observe that from any start point $n_2' \geq \frac{3L}{4}$ the expected time to reach $\frac{3L}{4}$ is L^2, and the probability that $\frac{3L}{4}$ is not reached within L^5 steps is exponentially small (Lemma S10). Hence if the probability to reach the target set from $\frac{3L}{4}$ within $p_1(L)$ steps is at least $\frac{1}{p_2(L)}$, then from all states the probability to reach within $L^5 \cdot p_1(L)$ steps is at least $\frac{1}{L \cdot p_2(L)}$. In other words, from any state the probability that the target set is not reached within $L^5 \cdot p_1(L)$ steps is at most $(1 - \frac{1}{L \cdot p_2(L)})$. Hence from any state the probability that the target set is not reached within $k \cdot L^5 \cdot p_1(L)$ steps is at most $(1 - \frac{1}{L \cdot p_2(L)})^k$. Thus from any state the probability that the target set is not reached within $L^3 \cdot p_2(L) \cdot L^5 \cdot p_1(L)$ steps is at most

$$\left(1 - \frac{1}{L \cdot p_2(L)}\right)^{L \cdot p_2(L) \cdot L^2} = e^{-L^2}.$$

Hence the probability to reach the target within $L^8 \cdot p_1(L) \cdot p_2(L)$ steps is at least $1 - \frac{1}{e^{L^2}}$. By Lemma S11 the expectation contribution from steps at least $L^2 \cdot 2^{L \cdot \log L}$ is constant ($O(1)$).

Hence we would obtain an upper bound on the expected hitting time as

$$L^8 \cdot p_1(L) \cdot p_2(L) \cdot \left(1 - \frac{1}{e^{L^2}}\right) + \frac{L^2 \cdot 2^{L \cdot \log L}}{e^{L^2}} + O(1) \le L^9 \cdot p_1(L) \cdot p_2(L).$$

Note that the above bound is obtained without assuming that $p_1(\cdot)$ and $p_2(\cdot)$ are polynomial functions. However, if $p_1(\cdot)$ and $p_2(\cdot)$ are polynomial, then we will obtain a polynomial upper bound on the hitting time, which contradicts the exponential lower bound. The desired result follows. □

Corollary S3. *In all cases, where the lower bound on the expected hitting time is exponential, let us denote by h denote the expected hitting time. Given numbers t_1 and t_2, the probability to reach the target set from any state n_2 such that $n_2 \ge \frac{3L}{4}$ within the first $t_1 = \frac{h}{L^9 \cdot t_2}$ steps is at most $\frac{1}{t_2}$.*

Proof. In the proof of Lemma S12 first we established that the hitting time is at most $L^9 \cdot p_1(L) \cdot p_2(L)$ (without assuming they are polynomial). By interpreting t_1 as $p_1(L)$ and $t_2 = p_2(L)$ we obtain that $h \le L^9 \cdot t_1 \cdot t_2$. The desired result follows. □

Theorem S5. *In all cases, where the lower bound on the expected hitting time is exponential, for all polynomials $p_1(\cdot)$, $p_2(\cdot)$ and $p_3(\cdot)$, for $p_3(L)$ independent multiple searches, the probability to reach the target set from any state n_2 such that $n_2 \ge \frac{3L}{4}$ within first $p_1(L)$ steps for any of the searches is at most $\frac{1}{p_2(L)}$.*

Proof. Consider the polynomial $\overline{p}_2(L) = p_3(L) \cdot p_2(L)$. Then by Lemma S12 for a single search the probability to reach the target within $p_1(L)$ steps is at most $\frac{1}{\overline{p}_2(L)}$. Hence the probability that none of the search reaches the target in $p_1(L)$ steps is

$$\left(1 - \frac{1}{\overline{p}_2(L)}\right)^{p_3(L)} = \left(1 - \frac{1}{\overline{p}_2(L)}\right)^{p_3(L) \cdot \frac{p_2(L)}{p_2(L)}} = \left(1 - \frac{1}{\overline{p}_2(L)}\right)^{\overline{p}_2(L) \cdot \frac{1}{p_2(L)}} = e^{-\frac{1}{p_2(L)}} \le 1 - \frac{1}{2 \cdot p_2(L)};$$

since $e^{-2 \cdot x} \le 1 - x$, for $0 \le x \le \frac{1}{2}$. The desired result follows. □

Remark S3. Observe that in Theorem S5 the independent searches could start at different starting points, and the result still holds, because in all cases we established an exponential lower bound, the lower bound holds for all starting points outside the target region.

8.2. Probability of hitting in a given number of steps. We now present a simple (and informal) argument for the approximation of the probability that none of M independent searches succeed to discover the target in a given number of b steps, where the expected discovery time for a single search is d, for $b << d$. The steps of the argument are as follows:

1. First we observe that the expected discovery time is the expected hitting time in a Markov chain, and the probability distribution of the hitting time in a Markov chain is largely concentrated around the expected hitting time d, when the expected hitting time is exponential and the starting state is far away from the target set. The result of sharp concentration around the expected hitting time is a generalization of the classical Chernoff bound for the sum of independent variables: the generalization for Markov chains is obtained by considering Azuma-Hoeffding's s inequality for bounded martingales [1, Chapter 7] that shows exponential concentration around the expected hitting time. Note that for Markov chains, the martingale for expected hitting time is bounded by 1 (as with every step the hitting time increases by 1).

2. Given that the probability distribution is concentrated around the mean, an approximation of the probability that a single search succeeds in b steps is at most $\frac{b}{d}$, for $b << d$.

3. Given independent events $\mathcal{E}_1, \mathcal{E}_2, \ldots, \mathcal{E}_M$ such that the success probabilities are $a_1, a_2, \ldots, a_M$, respectively, by independence (that allows us to multiply probabilities) the probability that none of the events succeed is $(1 - a_1) \cdot (1 - a_2) \ldots (1 - a_M)$. Hence for independent M searches the probability that none of the searches succeed in b steps when the expected hitting time for a single search is d is at least

$$\left(1 - \frac{b}{d}\right)^M = e^{-\frac{M \cdot b}{d}}.$$

The above reasoning gives an informal argument to obtain an upper bound on the probability of success of M independent searches in b steps.

9. Distributed Targets

We now discuss several cases of distributed targets for which the exponential lower bounds can be obtained from our results. We discuss the results for four letter alphabet.

1. Consider the example of distributed targets where the letters in a given L_0 number of positions are immaterial (e.g., the first four positions, the tenth position and the last four positions are immaterial, and hence $L_0 = 9$ in this case). Then we can simply apply our results ignoring the positions which are immaterial, i.e., the string space of size $L - L_0$, and apply all results with effective length $L - L_0$.
2. Consider the example where the target set is as follows: instead of the target of all σ's (i.e., $t = \sigma^L$), the target set has all sequences that have at least an $\alpha \cdot L$ length segment of σ's, for $\alpha > 1/2$. Then all the targets have an overlapping segment of $(2 \cdot \alpha - 1) \cdot L$ number of σ's from position $(1 - \alpha) \cdot L$ to $\alpha \cdot L$. We can then obtain a lower bound on the discovery time of these targets by considering as target set the superset containing all sequences with σ's in that region. In other words, we can apply our results with single target but the effective length is $(2 \cdot \alpha - 1) \cdot L$. A pictorial illustration of the above two cases is shown in Supplementary Figure 6.
3. We now consider the case of distributed targets that are chosen uniformly at random and independently, and let $m << 4^L$ be the number of distributed targets. Let the selection gradient extend up to a distance of cL from a target, for $c < 3/4$. Formally we consider any fitness landscape f that assigns zero fitness to a string whose Hamming distance exceeds cL from every target. We consider a starting sequence for the search and argue about the estimate on the expected discovery time.
   - First we consider the Markov chain M defined on $B(L)$ where every string s in $B(L)$ is a state of the Markov chain. The transition probability from a string s to a neighboring string in $\mathsf{Nh}(s)$ of Hamming distance 1 is $\frac{1}{|\mathsf{Nh}(s)|}$. The Markov chain M has the following two properties: it is (i) *irreducible*, i.e., the whole Markov chain M is a recurrent class; and (ii) *reversible*, i.e., if there is a transition probability from s to s', there is also a transition probability from s' to s.
   - Since M is irreducible and reversible, and due to its symmetric nature, it has a very fast *mixing time* (the number of steps required to converge to the stationary distribution). In particular, the stationary distribution, which is the uniform distribution over $B(L)$, is converged to with in $O(L \cdot \log L)$ steps [2].
   - Since $c < 3/4$, the expected time to reach a string from where the selection gradient to a specific target is felt is exponential (by Corollary S2). Thus given $m << 4^L$ and $c < 3/4$, a string from where the selection gradient to any target is felt is reached with in the first $O(L \cdot \log L)$ steps with low probability.
   - Since any string from where the selection gradient is felt to a target is reached with in the first $O(L \cdot \log L)$ steps with low probability, and after $O(L \cdot \log L)$ steps M converges to the uniform distribution, a lower bound on the expected discovery time can be obtained as follows: consider the probabilistic process that in every step chooses a string in $B(L)$ uniformly at random and the process succeeds if the chosen string has a Hamming distance at most cL from any of the target sequence. The expected number of steps required for the success of the probabilistic process is a

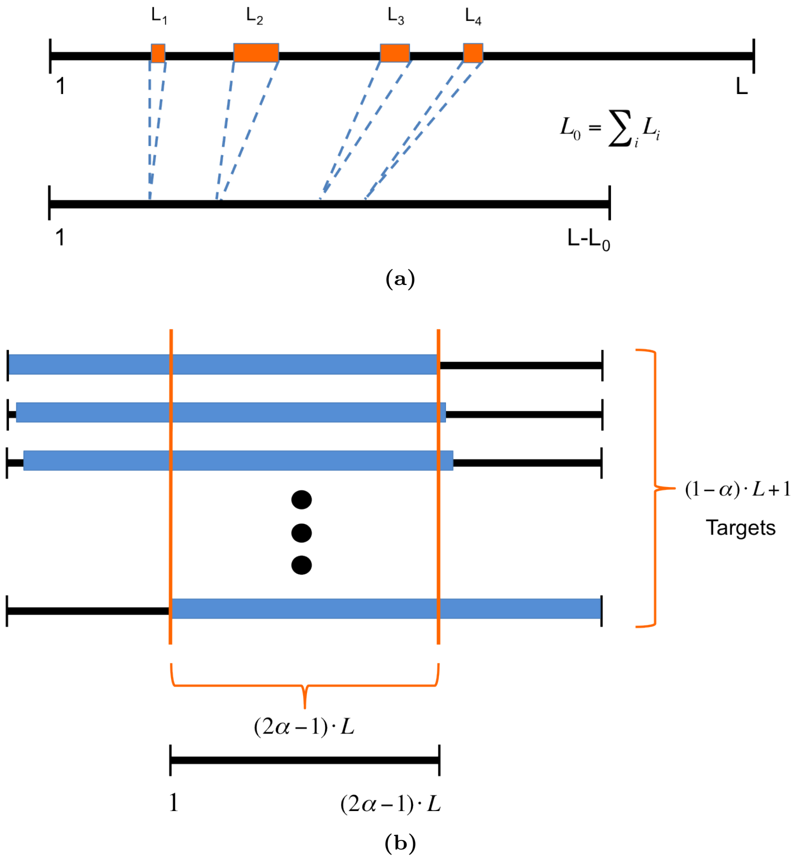

Supplementary Figure 6: Distributed target examples. Figure (a) shows that if there are positions of the string that are immaterial, then the effective length decreases. Figure (b) considers the case when the evolutionary process searches for a string of length $\alpha \cdot L$, and it shows that it searches for a single string of length at least $(2 \cdot \alpha - 1) \cdot L$.

lower bound on the expected discovery time. Hence we first estimate the success probability of every step for the probabilistic process. Consider a target string and a string chosen uniformly at random. Since the string is chosen uniformly at random, we can equivalently think that the process is generating uniform distribution over the alphabet for every position of the string sequence. The probability that the i-th position of the sequence of a target differs from the chosen sequence has probability $3/4$ (since we have a four letter alphabet). In other words, the generation of the positions of the string are Bernoulli random variables with mean $3/4$. Let X denote the random variable for the number of positions of a target that differ from the chosen sequence (in other words, X denotes the Hamming distance), and hence X is distributed according to *Bionomial*$(L, 3/4)$. We now apply Hoeffding's inequality and obtain that the probability that chosen string lies within the selection gradient from a specific target is at most

$$\mathbb{P}[X \leq cL] \leq \exp\left(-2 \cdot (3/4 - c)^2 \cdot L\right)$$

By union bound, the probability of success in every step is at most $m \cdot \exp\left(-2 \cdot (3/4 - c)^2 \cdot L\right)$, and thus the expected discovery time is at least $\frac{\exp\left(2 \cdot (3/4 - c)^2 \cdot L\right)}{m}$. Note that in proof of the lower bound above any sequence with positive fitness is considered as a target, and hence the lower bound on the expected discovery time holds even if there is a broad peak of width cL around each of the m target sequences.

Theorem S6. *Consider the four letter alphabet, and a starting sequence in $B(L)$. Let the target set of $m << 4^L$ sequences be chosen uniformly at random, with selection extending up to a distance of cL from each target sequence, with $c < 3/4$. Then with high probability the expected discovery time of the target set is at least* $\frac{\exp\left(2 \cdot (3/4 - c)^2 \cdot L\right)}{m}$.

Hence, if m is polynomial, or even an exponential smaller than $\exp\left(2 \cdot (3/4 - c)^2 \cdot L\right)$, then the expected discovery time is exponential with high probability.

10. A Mechanism for Polynomial Time

In the previous sections we have shown the scenarios where the discovery time is not polynomial. We now discuss a way that can ensure polynomial bounds. In the *regeneration process*, the process of evolution keeps on generating strings close to the target (say of distance k from the target). If the initial distance is k and constant, then we show with very high probability in polynomially many regenerations the target is reached.

Regeneration process. Formally, the regeneration process has two key aspects: (1) The process keeps on generating starting sequences; and (2) the starting sequences that are generated are only a constant number k steps away from some sequence in the target set.

Polynomially many regenerations. First note that from every string s, if there is a transition from the string s to a neighbor $\mathsf{Nh}(s)$, then there is at least a probability of $\frac{1}{4 \cdot L}$ to a neighbor that is closer to the target set. Hence in every step, if there is a mutation, then the probability to move closer to the target set is at least $\frac{1}{4 \cdot L}$ (and in expectation a transition to a neighbor occurs in every $\frac{1}{u}$ steps). Thus with probability at least $\alpha = \left(\frac{1}{4 \cdot L}\right)^k$ the target is reached for a single trial in $O(\frac{k}{u})$ steps, i.e., the probability is obtained as the product of the probabilities that each of the k steps of mutations bring closer to the target. Thus the probability to not reach in $L \cdot (4 \cdot L)^k = L \cdot \frac{1}{\alpha}$ independent trials (or regeneration steps) is at most

$$(1 - \alpha)^{\frac{1}{\alpha} \cdot L} = e^{-L};$$

i.e., exponentially small in L. In other words, with $L \cdot (4 \cdot L)^k = L \cdot \frac{1}{\alpha}$ trials (regenerations) the target is discovered in time at most $O(\frac{k}{u} \cdot L \cdot \frac{1}{\alpha})$ with very high probability; i.e., if k is constant, then with polynomially many regenerations the target is discovered in polynomial time with very high probability.

11. Calculations and Details of Data of Article

We first present a calculation of the number of targets in a broad peak.

Calculation 1. For a four letter alphabet, the number of sequences that differ in at most cL positions form $\vec{0}$ is

$$\sum_{i=0}^{cL} 3^i \binom{L}{i} \geq 3^{cL} \binom{L}{cL} = 3^{cL} \frac{L!}{(cL)!((1-c)L)!}$$

By Stirling's approximation we have $n! \geq \frac{\left(\frac{n}{e}\right)^n}{\sqrt{2\pi n}}$ and thus we have

$$\begin{aligned} \frac{L!}{(cL)!((1-c)L)!} &\simeq \frac{\left(\frac{L}{e}\right)^L \sqrt{2\pi L}}{\left(\frac{cL}{e}\right)^{cL} \sqrt{2\pi cL} \left(\frac{(1-c)L}{e}\right)^{(1-c)L} \sqrt{2\pi(1-c)L}} \\ &= \frac{1}{c^{cL+0.5}(1-c)^{(1-c)L+0.5}\sqrt{2\pi L}} \geq \frac{(1-c)^{(c-1)L}}{\sqrt{2\pi L}} \end{aligned}$$

where we first apply Stirling's approximation, and for the inequality use that since $c < \frac{3}{4}$ we have $\frac{1}{c^{cL+0.5}} \geq 1$. By converting the exponential to base 2 we obtain

$$\frac{(1-c)^{(c-1)L}}{\sqrt{2\pi L}} = \frac{2^{(c-1)L\log_2(1-c)}}{\sqrt{2\pi L}} \geq \frac{2^{c(1-c)L}}{\sqrt{2\pi L}}$$

since $-\log_2(1-c) \geq c$ for $0 \leq c < \frac{3}{4}$. Hence the number of sequences at hamming distance at most cL from $\vec{0}$ grows exponentially, as

$$\frac{3^{cL}2^{c(1-c)L}}{\sqrt{2\pi L}} \geq \frac{2^{cL}}{\sqrt{2\pi L}} \geq \frac{2^{cL}}{3L}$$

as $3^{cL} \geq 2^{c^2L}$ since $c < 1$, and $\sqrt{2\pi L} < 3L$.

Calculation 2. For $L = 100$ and $c = 0.10$, the number of strings in the cloud around the target is as follows:

$$\sum_{i=1}^{10} \binom{100}{i} \cdot 3^i \approx 1.06 \cdot 10^{18}$$

Calculation 3. We now apply the approximation results of Section 8.2 for calculation of success probability for multiple searches to discover the target in bounded number of steps. Let us consider $b = 10^{14}$ steps (upper bound for 4 billion years), and $M = 10^{24}$ independent searches, and let $L = 1000$ and $c = 1/2$. Then the expected discovery time for a single search is at least 10^{65}. Thus applying the formula $(1 - b/d)^M$ for the probability that none of the searches succeed we have the probability of failure for all searches is:

$$\left(1 - \frac{10^{14}}{10^{65}}\right)^{10^{24}} = \left(1 - \frac{1}{10^{51}}\right)^{10^{24}} = \left(1 - \frac{1}{10^{51}}\right)^{10^{51}\cdot 10^{-27}} \simeq \left(\frac{1}{e}\right)^{10^{-27}} = \frac{1}{\sqrt[10^{27}]{e}} \geq 1 - 10^{-26}.$$

Thus the probability of at least one search succeeding within 10^{14} generations is at most $10^{-26} \simeq 0$.

More precise version of Table 1 of article. A more elaborate and precise version of Table 1 of article is given below.

$r = 1$	$c = \frac{1}{3}$	$c = \frac{1}{2}$	$c = \frac{3}{4}$
$L = 10^2$	$1.027309 \cdot 10^{18}$	$7.366173 \cdot 10^{7}$	182.71
$L = 10^3$	$5.891566 \cdot 10^{170}$	$1.285790 \cdot 10^{65}$	2666.2

Supplementary Table 2: Table 1 of main text with higher precision. Table of numerical data for discovery time.

12. Related Work

In this section we discuss and compare our results with relevant related works from population genetics.

Genetic adaptation on continuous and sequence space. The subject of genetic adaptation has been an active research area for several decades, and has been nicely summarized by Orr [3]. In a seminal work [4], Fisher introduced the geometric model of adaptation in order to capture the statistical properties of beneficial mutations and their effect in a continuous phenotypic space. He concluded that evolution proceeds via mutations of small effect, a view that was first reconsidered later by Kimura [5]. Orr [6] extended this work of Kimura by studying the distribution of sizes of mutations for the whole evolutionary walk, and showed that it is an exponential distribution which retains its shape (but gradually shrinking) for the whole of the walk (also see [7] for a review and summary of this work). Kimura is also known for having introduced the neutral theory of molecular evolution [8]. To quote from Orr [3] "Throughout the 1960s and 1970s, evolutionary geneticists grew increasingly convinced that much, if not most, molecular evolution reflects the substitution of neutral [8,9] or nearly neutral [5,10–12] mutations, not beneficial ones." In [13, 14], Maynard Smith conceived the idea that organisms evolve in the discrete, high-dimensional space of DNA and protein sequences, and the adaptive walk proceeds via unit mutational steps to fitter sequences. The idea of exploring sequence space was expanded in [15], where evolution in rugged fitness landscapes was captured by the NK model. Recently, it has been demonstrated empirically that the ruggedness of the NK model is correlated, so that typically large peaks are clustered together [16]. Gillespie [17] described a simple stochastic substitution model under strong selection and weak mutation, and by means of extreme value theory concluded that the mean number of gene substitutions until fixation is small. This view was further developed in [18, 19], where the assumption that the starting sequence must be highly fit was necessary for efficient evolution. In a similar setting, Orr [20] showed that finding local optima in sequence spaces takes at least $e - 1$ steps where $e = 2.71$. This came as a conclusion from the observation that the mean distance to a local maxima is $e - 1$.

The speed of adaptation has also frequently been characterized in terms of fixation rates of beneficial mutations. Orr [21] studied the rate of adaptive substitutions in asexuals as a function of the mutation rate under the assumption that selection against the deleterious mutations is stronger than selection in favor of the beneficial one. It was shown that the mutation rate which maximizes the adaptation rate depends only the strength of selection against deleterious mutations. This work was later extended in [22] where it was shown that beneficial alleles with relatively small beneficial advantage also have relatively small probability of fixation.

Role of recombination. A key research question is what phenomenon contributes to speed-up of the evolutionary search process. The classical work of Crow and Kimura shows that recombination leads to a speed in evolution. Crow and Kimura [23] studied the advantage that recombination confers to an adapting sexual population over its asexual counterpart, by eliminating the clonal interference between simultaneously emerging beneficial mutants. In [23] the length L of the genome sequence is not a parameter, and the results show that the speed-up due to recombination is proportional to the population size. The speed-up of recombination in various models with L also as a parameter was considered by Maynard Smith, and

Table 1 in [24] summarizes the relative speed-up under various models. In the best case, the speed-up due to recombination is proportional to the product of the population size and the length L of the genome. Charlesworth in [25] also examined the advantage in the population mean fitness that sexual populations have over asexuals, for various dynamic selection functions, and showed that this advantage is substantial for various breeding systems. The advantage of sexual reproduction continuous to be an active topic of study. A recent study [26] used a theoretical argument to demonstrate that in infinite populations of two loci that can accumulate infinite many mutations each, as long as the rate of recombination is $r > 0$, the speedup advantage of the sexual populations over asexuals is approaches 2. This is because in infinite populations, no matter how small the recombination rate is, as long as it is non-zero, a small fraction of highly fit individuals due to recombination will always exist. Because this portion of the population has an exponentially large fitness advantage, it will spread fast, and the rate of adaptation approaches that of extreme recombination ($r = 1$), which was proven to be 2. In [27], several different modes of recombination were examined and it was shown that in all models, when the population size is large, the rate of adaptation has a logarithmic dependency on $N \cdot u$ (population size times beneficial mutation rate), rather than linear. However, the rate of adaptation has a quadratic dependency on the recombination rate, showing that recombination plays a more important role in this rate for large populations. There are, of course, other parameters that affect the rate of adaptation in sexuals. In [28], the authors examined sexual reproduction in chromosomes where short stretches are linked together, and hence, clonal interference exists locally. The population can be effectively seen as consisting of many asexual stretches linked together by a smaller recombination factor, and it was estimated that beneficial mutations fix at a rate proportional to the new rate of recombination, with only logarithmic dependency on the rate in which beneficial mutations are introduced.

Our results. In this work our contributions are as follows:

1. We present the mathematical foundations to estimate the expected number of steps for evolutionary processes as a function of L;
2. we characterize scenarios when the expected time is exponential in L;
3. we present strong dichotomy results between exponential vs polynomial time;
4. we suggest a mechanism that enables to break the infeasible exponential barrier and allows evolution to work in polynomial time.

In part, our work systematically explores fitness landscapes with large neutral regions, where broad peaks are distributed at random. Such landscapes are widespread in biology. Studies have shown that the fitness landscapes of RNA are typically highly neutral, with very low peak density (around 10^{-13}, according to [29]). Wagner's studies have also identified neutrality as an important ingredient for robustness [30, 31]. Elaborate computer simulations have also outlined the importance of random drift in the emergence of novel functions [32].

Our results nicely combine and explain several existing results. The regeneration process that breaks the exponential barrier requires that (i) the starting sequence starts only a constant number of steps away from the target and (ii) the starting sequence can be repeatedly generated. The first aspect is related to the results of Gillespie [18, 19] that using extreme value theory suggests that the starting sequence must be a highly fit sequence for efficient evolution (i.e., in our setting close to the target). The second aspect ties in with the long-standing ideas that gene (and genome) duplications are the major events leading to the emergence of new genes [33] and that evolution is a 'tinkerer' playing around with small modifications of existing sequences rather than creating entirely new sequences [34]. Recent studies [35] have shown that large numbers of transcirption factor binding sites have been evolved under divergent evolution, supporting the claim of local sequence duplication as a mechanism to emerge new functions. Our work shows that the combination of these two ideas break the exponential barrier. Our results also nicely combine with the existing results on recombination. Recombination that may lead to a linear factor speed-up does not change an exponential function to a polynomial one, but may contribute greatly to the efficiency of a polynomial process. The polynomial upper bound of L^{k+1} for regeneration process holds without selection and recombination. But the polynomial bound of L^{k+1} can still be inefficient, and then selection and possibly recombination plays the role to make the feasible polynomial bound much more efficient.

13. Additional Simulation Results

In this section we describe some additional computer simulation results. Our first simulation result is for the Moran process and per-bit mutation rate. The second simulation result is for another classical evolutionary process, namely, the Wright-Fisher process. The details are described in Supplementary Figure 7 and Supplementary Figure 8, respectively.

A. Technical Appendix: Linear Fitness Transition Probabilities

We derive the transition probabilities of the corresponding Markov chain on a line $M_{L,\beta}$ for the case of the linear fitness landscape and any selection intensity. That is, given s and $s' \in \mathsf{Nh}(s)$, we have $df(s,s') = (f(s') - f(s)) = -(h(s') - h(s))$. Our goal is to show that for $0 < i < L$ we have:

$$(i)\ \delta_\beta(i,i+1) = \frac{1}{1+e^{\beta}\cdot\frac{i}{L-i}} \qquad (ii)\ \delta_\beta(i,i-1) = \frac{1}{1+e^{-\beta}\cdot\frac{L-i}{i}}$$

For s in equivalence class $0 < i < L$, i.e. $h(s) = i$, there exist exactly i neighbors $s' \in \mathsf{Nh}(s)$ with $h(s') = i-1$ and $df(s,s') = 1$, and $L-i$ neighbors $s'' \in \mathsf{Nh}(s)$ with $h(s'') = i+1$ and $df(s,s'') = -1$. Then from the normalized sum of Eqn 1 we obtain:

(i)

$$\delta_\beta(i,i+1) = \frac{\frac{L-i}{1+e^{\beta}}}{\frac{L-i}{1+e^{\beta}} + \frac{i}{1+e^{-\beta}}} = \frac{1}{1+\frac{i}{L-i}\cdot\frac{1+e^{\beta}}{1+e^{-\beta}}} = \frac{1}{1+\frac{i}{L-i}\cdot e^{\beta}\cdot\frac{e^{-\beta}+1}{1+e^{-\beta}}} = \frac{1}{1+e^{\beta}\cdot\frac{i}{L-i}}$$

(ii) Let $x = e^{\beta}\cdot\frac{i}{L-i}$

$$\delta_\beta(i,i-1) = 1 - \delta_\beta(i,i+1) = 1 - \frac{1}{1+x} = \frac{x}{1+x} = \frac{1}{\frac{1}{x}+1} = \frac{1}{1+e^{-\beta}\cdot\frac{L-i}{i}}$$

because $\frac{1}{x} = e^{-\beta}\cdot\frac{L-i}{i}$.

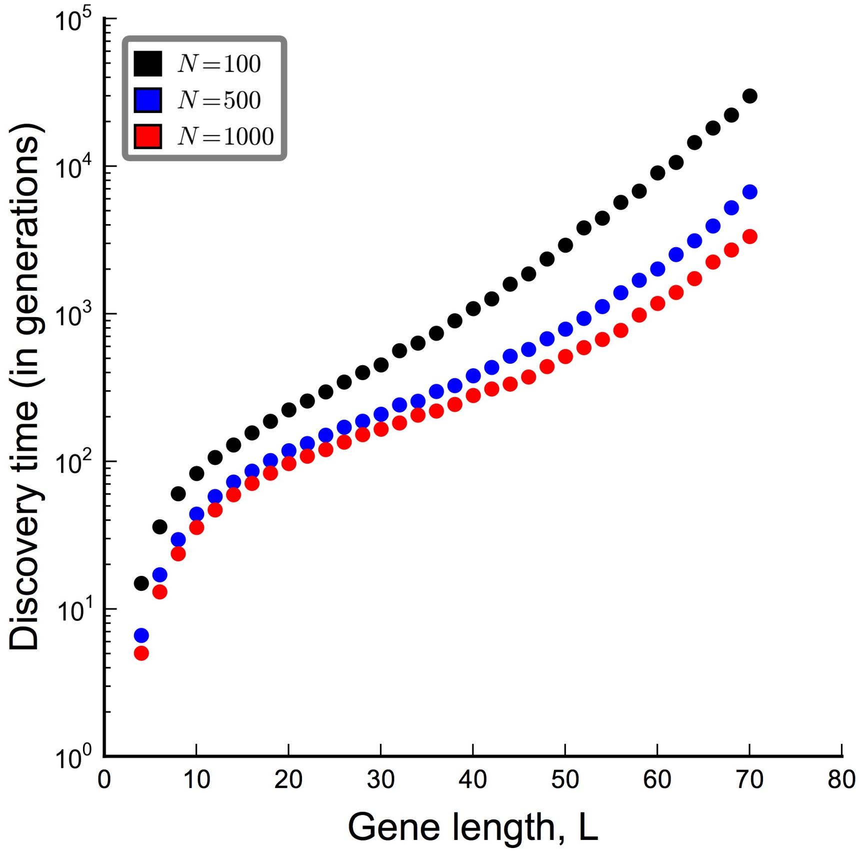

Supplementary Figure 7: Moran process with per-bit mutation rate. The figure shows the results of the average discovery time obtained from computer simulation of a Moran process with per-bit mutation rate. We consider the case of neutral drift with broad peak of $c = 1/2$. We consider a population of size N, and in each round an individual A is chosen at random to reproduce, and the off-spring A' of A is produced from the string of A with per-bit mutation rate of 1%. Then an individual is chosen at random to die and the off-spring A' replaces the dead individual (thus population size remains constant). The process stops as soon as one individual reaches a string with Hamming distance at most cL from the target (one individual hits the broad peak). The discovery time is the number of generations (reproductions) required by the individual who reaches the peak the first time. We ran the computer simulation for 1000 samples for each experiment and then plot the average discovery time, and the figure shows the result for $N = 100$, 500, and 1000, and shows the average discovery time as a function of the gene length L. We again observe that the discovery times grow exponentially in n in all cases.

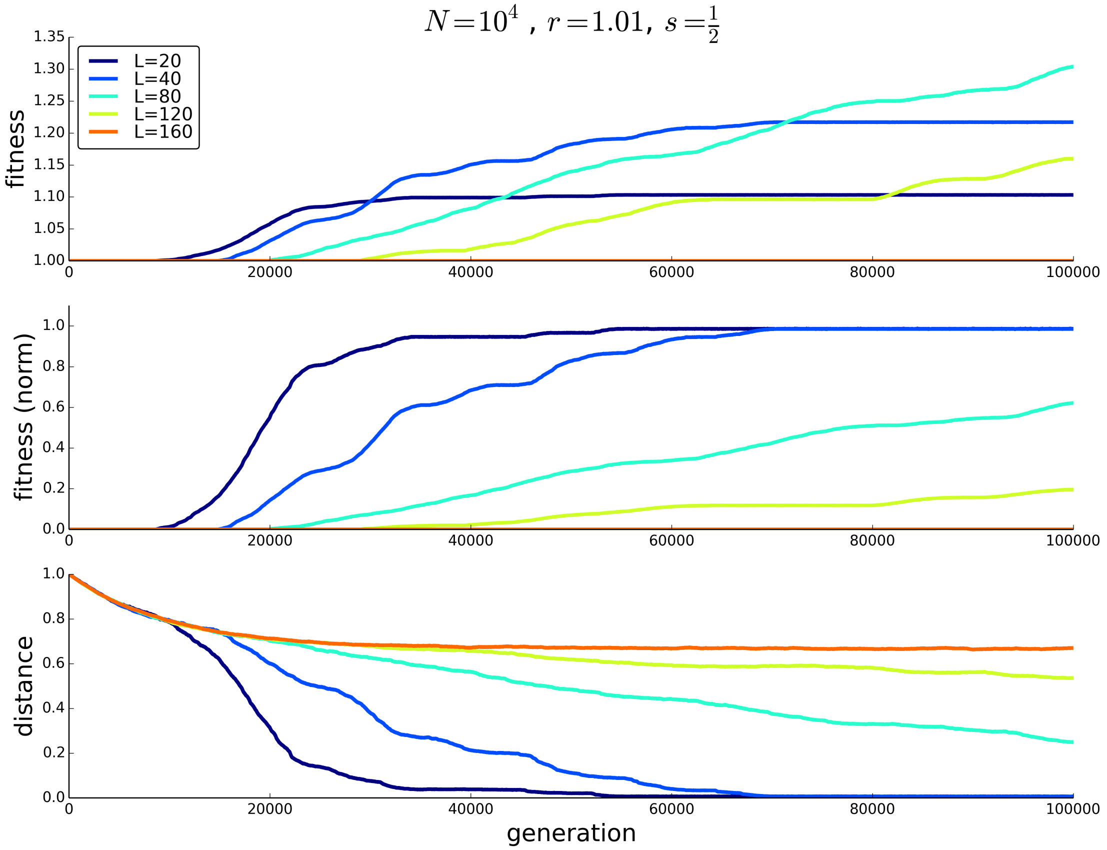

Supplementary Figure 8: Wright-Fisher Process. The figure shows the evolution of populations in the Wright-Fisher model, for population size $N = 10^4$, for various values of L. We consider the multiplicative fitness landscape with $r = 1.01$, and the selection is felt from $L/2$ away from the ideal sequence $\vec{0}$. At every generation a new population replaces the old one, such that the expected number of off-springs of an individual of the old population to the new one is proportional to its fitness. These off-springs are mutated with a uniform mutation rate per bit ($u = 10^{-4}$)). The first two figures depict the evolution of the mean fitness and normalized mean fitness of the population, while the last figure depicts the normalized average distance of the population from the target sequence $\vec{0}$. The results are obtained from a computer simulation where for each value of L the simulation was ran for 50 cases, and the averages are shown.

References

[1] Noga Alon and Joel H Spencer. *The probabilistic method.* New York: Wiley-Interscience, 2000.

[2] Persi Diaconis. *Group representations in probability and statistics.* Lecture Notes–Monograph Series, Volume 11, Institute of Mathematical Statistics, 1988.

[3] H. Allen Orr. The genetic theory of adaptation: a brief history. *Nat Rev Genet*, 6(2):119–127, Feb 2005.

[4] Ronald Aylmer Fisher. *The Genetical Theory of Natural Selection.* Clarendon Press, 1930.

[5] Motoo Kimura. *The Neutral Theory of Molecular Evolution.* Cambridge University Press, 1983.

[6] H. Allen Orr. The population genetics of adaptation: The distribution of factors fixed during adaptive evolution. *Evolution*, 52(4):935–949, 1998.

[7] Nick Barton. Evolutionary biology: The geometry of adaptation. *Nature*, 395(6704):751–752, 1998.

[8] Motoo Kimura. Evolutionary rate at the molecular level. *Nature*, 217:624–626, 1968.

[9] Jack Lester King and Thomas H. Jukes. Non darwinian evolution: random fixation of selectively neutral mutations. *Science*, 164:788–798, 1969.

[10] Tomoko Ohta. Molecular evolution and polymorphism. *(ed. Kimura, M) National Institute of Genetics, Mishima*, pages 148–167, 1977.

[11] Motoo Kimura. Model of effectively neutral mutations in which selective constraint is incorporated. *Proceedings of the National Academy of Sciences*, 76(7):3440–3444, 1979.

[12] T Ohta. The nearly neutral theory of molecular evolution. *Annual Review of Ecology and Systematics*, 23(1):263–286, 1992.

[13] John Maynard Smith. *The Scientist Speculates: an Anthology of Partly-Baked Ideas (ed Good, I.J.).* Basic Books, 1962.

[14] John Maynard Smith. Natural selection and the concept of a protein space. *Nature*, 225(5232):563–564, 1970.

[15] Stuart Kauffman and Simon Levin. Towards a general theory of adaptive walks on rugged landscapes. *Journal of Theoretical Biology*, 128(1):11 – 45, 1987.

[16] B. Østman, A. Hintze, and C. Adami. Critical properties of complex fitness landscapes. *ArXiv e-prints*, June 2010.

[17] John H. Gillespie. A simple stochastic gene substitution model. *Theoretical Population Biology*, 23(2):202 – 215, 1983.

[18] John H. Gillespie. Molecular evolution over the mutational landscape. *Evolution*, 38(5):1116–1129, 1984.

[19] J H Gillespie. Natural selection and the molecular clock. *Molecular Biology and Evolution*, 3(2):138–155, 1986.

[20] H. Allen Orr. A minimum on the mean number of steps taken in adaptive walks. *Journal of Theoretical Biology*, 220(2):241 – 247, 2000.

[21] H. Allen Orr. The rate of adaptation in asexuals. *Genetics*, 155(2):961–968, 2000.

[22] Toby Johnson and Nick H. Barton. The effect of deleterious alleles on adaptation in asexual populations. *Genetics*, 162(1):395–411, 2002.

[23] James F. Crow and Motoo Kimura. Evolution in sexual and asexual populations. *The American Naturalist*, 99(909):pp. 439–450, 1965.

[24] J. Maynard Smith. Recombination and the rate of evolution. *Genetics*, 78(1):299 – 304, 1974.

[25] Brian Charlseworth. Directional selection and the evolution of sex and recombination. *Genetics Research*, 61:205–224, 1993.

[26] Su-Chan Park and Joachim Krug. Rate of adaptation in sexuals and asexuals: A solvable model of the fishermuller effect. *Genetics*, 195(3):941–955, 2013.

[27] R. A. Neher, B. I. Shraiman, and D. S. Fisher. Rate of adaptation in large sexual populations. *Genetics*, 184(2):467–481, 2010.

[28] Daniel B. Weissman and Oskar Hallatschek. The rate of adaptation in large sexual populations with linear chromosomes. *Genetics*, 196(4):1167–1183, 2014.

[29] Jos I. Jimnez, Ramon Xulvi-Brunet, Gregory W. Campbell, Rebecca Turk-MacLeod, and Irene A. Chen. Comprehensive experimental fitness landscape and evolutionary network for small rna. *Proceedings of the National Academy of Sciences*, 2013.

[30] S. Ciliberti, O. C. Martin, and A. Wagner. Innovation and robustness in complex regulatory gene networks. *Proceedings of the National Academy of Sciences*, 104(34):13591–13596, 2007.

[31] Andreas Wagner. Robustness and evolvability: a paradox resolved. *Proceedings of the Royal Society B: Biological Sciences*, 275(1630):91–100, 2008.

[32] RE Lenski, C. Ofria, R.T. Pennock, and C. Adami. The evolutionary origin of complex features. *Nature*, 423(6936):139–44, 2003.

[33] Susumu Ohno. *Evolution by gene duplication*. Springer-Verlag, 1970.

[34] F Jacob. Evolution and tinkering. *Science*, 196(4295):1161–1166, 1977.

[35] A. Nourmohammad and M. Lässig. Formation of regulatory modules by local sequence duplication. *PLoS Computational Biology*, 7:e1002167, 2011.
